# The Kidney Transcriptome and Proteome Defined by Transcriptomics and Antibody-Based Profiling

**DOI:** 10.1371/journal.pone.0116125

**Published:** 2014-12-31

**Authors:** Masato Habuka, Linn Fagerberg, Björn M. Hallström, Caroline Kampf, Karolina Edlund, Åsa Sivertsson, Tadashi Yamamoto, Fredrik Pontén, Mathias Uhlén, Jacob Odeberg

**Affiliations:** 1 School of Biotechnology, Science for Life Laboratory, KTH - Royal Institute of Technology, Stockholm, Sweden; 2 Department of Immunology, Genetics and Pathology, Science for Life Laboratory, Uppsala University, Uppsala, Sweden; 3 Department of Structural Pathology, Institute of Nephrology, Medical and Dental School, Niigata University, Asahimachi-dori Niigata, Japan; 4 Department of Medicine, Karolinska Institutet and Centre for Hematology, Karolinska University Hospital, Stockholm, Sweden; University of Glasgow, United Kingdom

## Abstract

To understand renal functions and disease, it is important to define the molecular constituents of the various compartments of the kidney. Here, we used comparative transcriptomic analysis of all major organs and tissues in the human body, in combination with kidney tissue micro array based immunohistochemistry, to generate a comprehensive description of the kidney-specific transcriptome and proteome. A special emphasis was placed on the identification of genes and proteins that were elevated in specific kidney subcompartments. Our analysis identified close to 400 genes that had elevated expression in the kidney, as compared to the other analysed tissues, and these were further subdivided, depending on expression levels, into *tissue enriched*, *group enriched* or *tissue enhanced*. Immunohistochemistry allowed us to identify proteins with distinct localisation to the glomeruli (n = 11), proximal tubules (n = 120), distal tubules (n = 9) or collecting ducts (n = 8). Among the identified kidney elevated transcripts, we found several proteins not previously characterised or identified as elevated in kidney. This description of the kidney specific transcriptome and proteome provides a resource for basic and clinical research to facilitate studies to understand kidney biology and disease.

## Introduction

The main function of the kidney is to regulate the composition of the blood i.e. the levels of water, electrolytes, buffers and small molecules. Furthermore, it has an important role in elimination of organic compounds, metabolic waste products and many pharmaceutical drugs. A detailed knowledge of the proteins expressed by the cells comprising the different parts of the kidney forms the basis for understanding their functions, under both physiological and pathological conditions. Previously, molecular biology studies have lead to the identification of many proteins important for kidney function. These include nephrin (NPHS1), a primary component of slit-membrane in the glomerulus that functions as the glomerular ultrafiltration barrier [1], aquaporin families (AQP) that are key molecules for water re-absorption in proximal tubules, descending limb of Henle's loop and collecting ducts [2] and many other transporters involved in the re-absorption/secretion of organic substrates or solutes [3] [4]. Takemoto *et al.* identified glomerulus transcripts in mouse kidney through large-scale sequencing and microarray profiling [5], while Miyamoto *et al.* identified the proteins localised in glomeruli using 2D SDS-PAGE and LC-MS/MS [6]. Despite these advances in our knowledge, a comprehensive kidney-specific transcriptome and proteome has not yet been defined.

We recently performed a large RNAseq analysis on 27 human tissues, covering all other major organs [7]. We have here used this data to define the kidney-specific transcriptome by comparing the kidney RNAseq analysis to that from the 26 other tissues [7]. This analysis was used as a basis for antibody-based staining for the proteins in kidney sections, using The Human Protein Atlas (www.proteinatlas.org) with more than 50,000 samples of kidney tissue analysed with immunohistochemistry and individually annotated by certified pathologists [8]. Thus, the transcriptomics analysis of kidney homogenate, with its mixture of cell types, was supplemented by immunohistochemistry analysis to determine the precise spatial distribution of the corresponding proteins. In this manner, we have generated a knowledge resource with a comprehensive list of genes elevated in kidney with data on specificity and localisation of the corresponding proteins in the various nephron segments of the kidney.

## Materials and Methods

### Sample characteristics

The tissue samples used for transcript profiling of human kidney included histologically normal tissue from operated material from four individuals: Female, 58 years (Sample 1); female, 67 years (Sample 2); female, 55 years (Sample 3); male 46 years (Sample 4).

The kidney tissue samples were collected from surgical specimens of resected kidneys from individuals operated for renal cell carcinoma. The tissue was sampled from the normal, healthy part of the kidney and was confirmed microscopically as having a normal morphology by a trained pathologist. The corresponding histology of each biopsy can be found in S1 Fig.

### Transcript profiling (RNA-seq)

The four individual kidney samples selected for RNA analysis comprise tissue from the cortex and medulla (S1 Fig.). The use of human tissue samples was approved by the Uppsala Ethical Review Board (Ups 02-577, no. 2011/473). Human tissue samples used for protein and mRNA expression analyses were collected and handled in accordance with Swedish laws and regulation and obtained form the Department of Pathology, Uppsala University Hospital, Uppsala, Sweden as part of the sample collection governed by the Uppsala Biobank (http://www.uppsalabiobank.uu.se/en/). All human tissue samples used in the present study were anonymised in accordance with approval and advisory report from the Uppsala Ethical Review Board (Dnr Ups 02-577 (protein) and Dnr 2011/473 (RNA)), and consequently the need for informed consent was waived by the ethics committee. The use and analyses based on human tissues has previously been described in Fagerberg L et al. [7]. Tissues samples were embedded in Optimal Cutting Temperature (O.C.T.) compounds and stored at −80°C. Hematoxylin-eosin (HE) stained frozen sections (4 µm) were prepared from each sample using a cryostat and the CryoJane Tape-Transfer System (Instrumedics, St. Louis, MO, USA). To ensure proper tissue morphology each slide was examined by a pathologist. From each frozen tissue block three sections (10 µm) were cut and collected for RNA extraction. Tissue was homogenised using a 3 mm steel grinding ball (VWR). Total RNA was extracted using the RNeasy Mini Kit (Qiagen, Hilden, Germany) according to the manufacturer's instructions. RNA samples were analysed using either an Agilent 2100 Bioanalyser system (Agilent Biotechnologies, Palo Alto, USA) with the RNA 6000 Nano Labchip Kit or an Experion automated electrophoresis system (Bio-Rad Laboratories, Hercules, CA, USA) with the standard-sensitivity RNA chip. All samples had an RNA integrity number of at least 7.5. mRNA sequencing was performed on Illumina HiSeq2000 and 2500 machines (Illumina, San Diego, CA, USA) using the standard Illumina RNA-seq protocol with a read length of 2×100 bases.

### Analysis of data

The software ‘Sickle’ (http://github.com/najoshi/sickle) was used to trim raw reads obtained from the sequencing system for low quality ends, using a phred quality threshold of 20. After discarding reads shorter than 54 bp, processed reads were mapped to the GRCh37 version of the human genome with Tophat v2.0.3 [9]. Potential PCR duplicates were eliminated using the MarkDuplicates module of Picard 1.77 (http://picard.sourceforge.net/). Quantification scores for all human genes, FPKM (fragments per kilobase of exon model per million mapped reads) values were calculated using Cufflinks v2.0.2 [10], which corrects for transcript length and the total number of mapped reads from the library to compensate for different read depths for different samples. The gene models from Ensembl build 69 were used in Cufflinks [11]. In addition to Cufflinks, HTSeq v0.5.1 was run to calculate read counts for each gene. These were used in the DESeq package for analyses of differentially expressed genes [12]. All data was analysed using R Statistical Environment (http://www.r-project.org/) with the addition of package ‘gplots’ (http://cran.r-project.org/web/packages/gplots/index.html). Network analysis was performed using Cytoscape 3.0 [13]. Where a log2-scale of the data was used for analyses, pseudo-counts of +1 were added to the data set. The significance of the enriched/enhanced genes were calculated using the DESeq software [14], doing a pairwise comparison between all the biological replicates of the enriched/enhanced tissue (or group of tissues) and all other tissues. The multiple-testing adjusted P-value (FDR 5%) was used to determine the significance of the enrichment.

### Specificity classification

An average FPKM value of all individual samples for each tissue was used to estimate the gene expression level. A cut-off value of 1 FPKM was defined as the detection limit. Each of the 20,050 genes were classified into one of seven categories based on the FPKM levels: (1) “Not detected” - <1 FPKM in kidney; (2) “Highly kidney enriched” - 50-fold higher FPKM level in kidney compared to all other tissues; (3) “Moderately kidney enriched” - 5-fold higher FPKM level in kidney compared to all other tissues; (4) “Group enriched” – 5-fold higher average FPKM level in a group of 2–7 tissues including kidney compared to all other tissues; (5) “Expressed in all tissues” – detected in 27 tissues>1 FPKM; (6) “kidney enhanced” – 5-fold higher FPKM level in kidney compared to the average FPKM value of all 27 tissues; (7) “Mixed” – genes expressed in 1–26 tissues and in none of the above categories.

### Tissue profiling

Tissue microarrays (TMA) containing biological triplicate 1-mm cores of 46 different types of normal tissue from different individuals and duplicate 1-mm cores of 216 different cancer tissues representing the 20 most common forms of human cancer were generated as previously described [15]. All of the tissues used as donor blocks were acquired from the archives at the Department of Pathology of Uppsala University Hospital in agreement with approval from the Research Ethics Committee at Uppsala University (Uppsala, Sweden)(Ups 02-577). TMA sections were immunostained as previously described [16]. The AperioScanScope XT Slide Scanner (Aperio Technologies, Vista, CA) system was used to capture digital whole slide images with a 20X objective. Slides were de-arrayed to obtain individual cores. The outcome of the TMA IHC stainings in the screening phase, was manually evaluated by certified pathologists and and scored using a web-based annotation system (unpublished). In brief, the manual score of IHC-based protein expression was determined as the fraction of positive cells defined in different tissues: 0 = 0–1%, 1 = 2–25%, 2 = 26–75%, 3>75% and intensity of immunoreactivity: 0 = negative, 1 = weak, 2 = moderate and 3 = strong staining. The immunohistochemical staining was visually evaluated with regard to kidney specificity and cellular distribution allowing further determination of protein localisation within different sub-compartments/cell types in the kidney tissue.

### Gene ontology analysis of nephron segment and collecting duct-specific proteomes

A gene ontology analysis [17] was performed using the GOrilla tool [18] in order to determine overrepresented GO categories for the different nephron segment specific proteomes we identified. For each nephron segment, GOSlim GOA were used to determine: 1) biological processes; 2) the molecular function; 3) sub-cellular localisation. The top 10 GO terms for each nephron segment and collecting duct were compared.

### Analysis of kidney enriched protein expression based on IHC annotations in the Human Protein Atlas database

We queried the Human Protein Atlas database for proteins that were annotated as expressed either uniquely in kidney, or expressed in kidney and not more than 6 other tissues (at lower or equal levels to that found in kidney). These criteria were chosen to correspond to criteria applied to the transcriptome datasets to identify genes classified into the ‘Group Enriched’ and ‘Kidney Enriched’ gene categories.

## Results

### Analysis of the kidney tissue transcriptome

**Figure 1:**
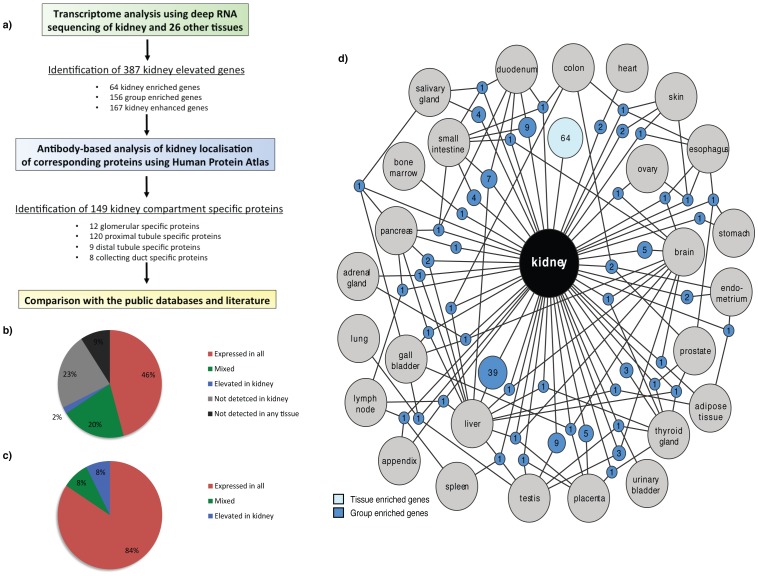
Correlation and classifications of genes expressed in kidney in relation to gene expression in other tissues. (a) Flow chart for the identification of kidney elevated genes and genes with unique localization to one nephron segment or collecting duct performed in this work. (b) Distribution of kidney expressed genes as the percentages of expressed mRNA molecules, i.e. the sum of all FPKM values for the genes expressed in kidney for each of the categories. (c) The classification of all human 20,050 protein coding genes divided in six different categories based on transcript abundance and number of detected tissues show 68% of human protein coding genes expressed in kidney. Colours in (b)-(c) represent six categories: not detected (grey), highly tissue enriched, moderately tissue enriched or group enriched (blue), tissue enhanced (dark blue), mixed (green), expressed in all low (red), expressed in all (dark red). (d) Network plot of the kidney enriched genes (light blue) and the group-enriched genes shared with ≤3 other tissues (dark blue). Blue circle nodes represent a shared group of expressed genes and are connected to the respective enriched tissues (grey circles). The size of each blue node is related to the square root of the number of genes enriched in a particular combination of tissues.

We have earlier described deep sequencing (RNAseq) analysis of 27 tissue types corresponding to all major tissues and organs in the human body, including four samples from kidney [7]. We used this as a platform to produce this comprehensive knowledge resource of location-specific kidney protein expression. The work-flow followed in the analysis is outlined in Fig. 1a.

Histological analyses of all the kidney specimens used in the study were performed to verify that cell type compositions of samples included were representative of the respective tissue (S1 Fig.). In the analysed kidney samples, the approximate constitution was: glomerular cells (10–26%), tubule cells (57–78%), endothelial cells (2–4%), fibroblasts (4–8%), inflammatory cells (4–10%) and smooth muscle cells (0–2%). The transcriptome of each sample was quantified to determine the normalised mRNA levels, calculated as FPKM (see Methods for details). The most abundantly expressed gene in the kidney (J01415.25) had a FPKM value of 15,484 and the number of genes that showed an expression value of>1 FPKM varied from 13,406 to 13,920. Thus, approximately 68% of all putative protein coding genes (n = 20,050) were detected in the kidney.

The variation between individual kidney samples were analysed through pairwise correlations, plotting the expression level (FPKM values) of all protein coding genes. A strong correlation between samples was found (Spearman correlations across all genes ranging between 0.96 to 0.98) illustrated with an example of pair-wise comparison of two individuals (S2 Fig.). The low inter-individual variation in genome-wide expression indicated high technical reproducibility and low biological variance. As expected, pairwise comparisons between the kidney and the 26 other tissue types showed a higher variance. The lowest correlation was found between kidney and testis (Spearman correlation of 0.69) while thyroid gland tissue displayed the highest similarity (0.88) to kidney (data not shown).

### Classification of the genes expressed in kidney

All putative protein coding genes (n = 20,050) were classified into four categories; (1) not detected in kidney (6,365 genes), (2) ‘kidney elevated’ (387 genes) - subdivided into ‘kidney enriched’ (expression level 5 times greater than any other tissue, respectively), group enriched (5-fold higher average expression in a group of 2-7 tissues including kidney, compared to all other tissues) or ‘kidney enhanced’ (expression level over 5 times the average across all other tissue types), (3) a mixed group of genes that were found to be expressed in several, but not all tissue types, and furthermore did not full-fill the criteria for tissue-elevated genes (4,048 genes), and (4) ‘housekeeping’ genes, defined as genes expressed in all 27 tissue types (9,250 genes) (Fig. 1b). Analysis of the expression levels of each gene in the kidney made it possible to calculate the relative mRNA pool for each of the categories as the total sum of FPKM values (Fig. 1c). 84% of the mRNA molecules in the kidney corresponded to ‘housekeeping genes’ and only 8% of the mRNA pool corresponded to genes categorised to be either kidney enriched, group enriched or enhanced. Thus, most transcriptional activity in the kidney is likely to involve ‘housekeeping’ functions. Among the 30 genes with the highest levels of expression in the kidney (S1 Table), only four were not ‘house-keeping’ genes - these were the kidney enriched gene uromodulin and three group enriched genes (ALDOB, SPP1, FXYD2). Uromodulin is expressed on the apical surface of the distal tubule and although a well-known kidney protein found to be the most abundant protein in the urine of healthy individuals, its specific function is still not fully understood [19]–[21].

64 genes were found to be tissue-enriched (Table 1 and S2 Table) and 167 genes were ‘kidney enhanced’ (S1 File). 156 group-enriched genes were found to have at minimum 5-fold higher average FPKM level in a group of 2–7 tissues including kidney compared to all other tissues analysed in the body (S2 File). The kidney shares the most group-enriched genes with the liver (Fig. 1d), and a more in-depth analysis of these genes (n = 39) showed that many of the corresponding proteins belong to the family of intracellular enzymes (S3 Table).

**Table 1:** The highly kidney-enriched genes according to the transcriptome profiling.

Gene name	Description	Main localisation	Kidney mRNA	TS score	HPA
UMOD	Uromodulin	Membrane	1421	647	Yes
SLC22A8	Solute carrier family 22, member 8	Membrane	269	633	Yes
SLC12A1	Solute carrier family 12, member 1	Membrane	406	460	Yes
TMEM174	Transmembrane protein 174	Membrane	97	443	Yes
MCCD1	Mitochondrial coiled-coil domain 1	Membrane	27	271	Yes
SLC34A1	Solute carrier family 34, member 1	Membrane	176	219	Yes
AQP2	Aquaporin 2 (collecting duct)	Membrane	315	177	Yes
SLC22A12	Solute carrier family 22, member 12	Membrane	108	162	Yes
SLC22A2	Solute carrier family 22, member 2	Membrane	97	154	Yes
SLC22A6	Solute carrier family 22, member 6	Membrane	198	144	No
KCNJ1	Potassium inwardly-rectifying channel	Membrane	132	140	Yes
SLC7A13	Solute carrier family 7, membrane 13	Membrane	19	118	No
NPHS2	Nephrosis 2, idiopathic, (podocin)	Membrane	68	116	Yes
SLC6A18	Solute carrier family 6, member 18	Membrane	11	106	Yes
SLC36A2	Solute carrier family 36, member 2	Membrane	64	97	Yes
SLC12A3	Solute carrier family 12, member 3	Membrane	129	84	Yes
SLC22A13	Solute carrier family 22, member 13	Membrane	12	74	Yes
ATP6V1G3	ATPase, H+ transporting, lysosomal	Membrane	38	73	Yes
MIOX	Myo-inositol oxygenase	Cytoplasm	625	73	Yes
TMEM207	Transmembrane protein 207	Membrane	10	59	Yes

‘Kidney mRNA’ shows the average FPKM value in the kidney samples, ‘TS’ shows the tissue specificity score and ‘HPA’ shows if there was corresponding data in the Human Protein Atlas. Individual FPKM values and variance in the four different kidney samples are found in S3 File.

### Antibody based profiling of the kidney specific genes

In order to validate the translation of the kidney-specific genes identified, immunohistochemistry was used to stain kidney sections from various individuals for the corresponding proteins. This data was generated within our Human Protein Atlas project (HPA), which currently comprises over 13 million images from stained tissue microarray (TMA) cores from 46 different normal tissues from different individuals. The RNAseq data and immunohistochemistry images for the kidney genes are publically available in the Human Protein Atlas resource (www.proteinatlas.org). The immunohistochemical staining was used to confirm kidney expression and to determinate protein localisation within different sub-compartments and cell types. Out of the 387 genes that we identified as elevated in kidney, the localisation of expression in kidney could be analysed using the HPA for 339 of the encoded proteins.

### Compartment specific localisation of protein expression

This list of identified genes is a valuable resource for the study of the molecular mechanisms of kidney biology and disease. Of particular importance, in order to evaluate their possible role in the kidney is the identification of the expression location(s). Out of the 387 genes identified as ‘kidney elevated’, 148 could be localised to only one sub-segment of the nephron or collecting duct (S4 Table). These were localised to glomeruli (11 proteins), proximal tubule (120 proteins), distal tubule (9 proteins), or collecting duct (8 proteins - of which 3 are expressed only in the subset of intercalated cells in collecting duct). Gene Ontology-based analysis of the 148 genes identified as specific for one segment only, is in agreement with the known functions in respective segment, such as glomeruli, proximal tubule, distal tubule and collecting duct (S5 Table).

#### Proximal tubule-specific proteins

**Figure 2:**
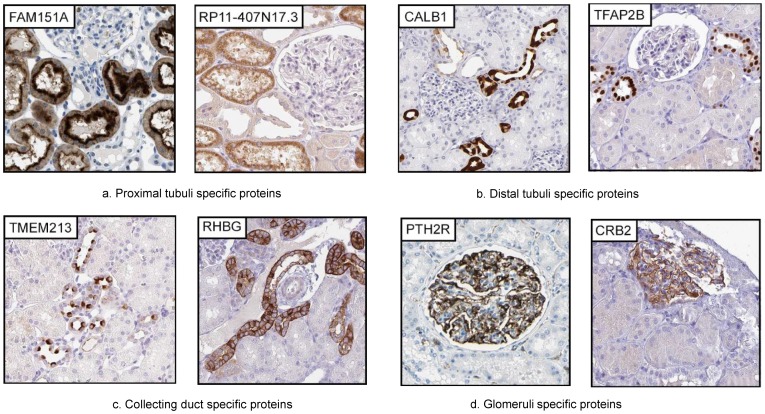
Proteins among the enriched/enhanced proteins localised to one specific nephron segment or collecting ducts. Two examples of proteins specific for each segment (or two segments) are shown, with segment name below images. (A) Among genes restricted to proximal tubule only, FAM151A is localised to luminal side while RP-11-407N17.3 show a cytoplasmic staining. (B) In distal tubule, CALB1 show a non-uniform cytoplasmic localisation, while TFAP2B show a distinct nuclear localisation in most cells. (C) In the collecting duct, TMEM213 is found at the luminal membrane of intercalated cells only, while RHBG display a general membrane localisation in all cells. (D) PTH2R and CRB2 show a specific localisation to podocytes in glomeruli.

Most of the ‘kidney elevated’ proteins (120) we identified were localised to the proximal tubule of which 59 had not been previously described in the context of proximal tubules (S4 Table). Our Gene Ontology analysis results are well in line with the function of proximal tubulus as a compartment for reabsorption of small molecules back to the blood. In particular, we identified 32 members of the solute carrier family proteins (SLC) [22]. Interestingly, for 3 of these (SLC16A9, SLC27A2, SLC5A11) the specific localisation within the kidney have not previously been described, and another 3 (SLC22A8, SLC28A1, SLC6A12) are not known to have an expression restricted to the proximal tubule, such as SLC6A12, which has previously been described as expressed in the collecting duct [23]. We identified three proteins (CRYAA, FAM151A and RP11-407N17.3) localised to the proximal tubule, not previously described as kidney expressed, neither in literature nor in public data repositories (Fig. 2a shows example staining). FAM151A was previously described to be present in plasma, urine, platelets and liver (http://www.genecards.org/), and RP11-407N17.3 has been described as a tumor associated antigen, cTAGE-5, found in cutaneous T-cell lymphoma and several other cancers [24], however the functions of both these genes are largely unknown. CRYYA, one of two alpha-crystallins, is a small heatshock protein whose only known function is in maintaining eye lens transparency, being a negative regulator of apoptosis, and *in vitro* it has been shown to trap aggregation-prone denatured proteins (for review see [25]). Mutations in this gene is linked to hereditary cataract formation [26]. The only other tissue we identified as an expression site was the liver (6.5 times lower), which is intriguing and indicates a specific, but currently unknown role for kidney (and liver) function.

#### Distal tubule-specific proteins

Among the 9 distal tubulus-elevated proteins (Table S4), the majority are membrane proteins with transport function (Fig. 2b), as exemplified by Calbindin1 (CALB1), a transporter already described in literature as localised to the distal tubulus [27]. In contrast, the transcription factor AP-2 Beta (TFAPB2), a member of the AP2 family of transcription factors, has not been previously described in the context of the adult kidney and here we show a distinct nuclear localisation, restricted to distal tubule cells (Fig. 2b). Interestingly, previous studies showed TFAP2B has an important role in embryonic development of the kidney and loss of this gene is associated with congenital polycystic kidney disease, due to excessive apoptosis of renal epithelial cells [28].

Other proteins we identified as distal tubulus located include several membrane proteins for electrolyte transport, including potassium, sodium and calcium transporters, consistent with the function of the distal tubule as site of electrolyte reabsorption and exchange between blood and urine [29]. In the distal tubulus we also identified Uromodulin, discussed above.

#### Collecting duct-specific proteins

Proteins we identified as kidney elevated with expression localised to the collecting ducts were mainly involved in water and proton transport, consistent with the function of collecting ducts in water re-absorption and control of pH homeostasis [30]. These include three trans-membrane proteins of the 8 proteins distinctly localised to the collecting duct – Aquaporin 2 (AQP2), the sodium bicarbonate transporter SLC4A9, and the ammonia transporter RHBG (Fig 2c). Three genes not previously described as localised to the collecting duct were identified (PVALB, CLNK, TMEM213). These displayed a localisation restricted to intercalated cells, in the case of TMEM213 with a polarised expression pattern towards apex (Fig. 2c).

#### Glomerular-specific proteins

We identified 11 glomerular elevated proteins (S4 Table) and the result of the analysis for these was well in line with the filtrating function of the glomerulus. The glomerulus enriched proteins we identified included PODXL and NPHS1, which have already been described as expressed here [31], [32]. These create the slit-membrane filtration barrier for large molecules together with NEPH1 (KIRREL) [33]. PTPPRO is a tyrosine phosphatase known to be unique for the podocyte and here we confirmed it to be highly expressed both on the RNA and protein levels in kidney, but our transcriptome data suggested that it is also expressed in the brain and in colon. We could identify one novel kidney enhanced protein localised to glomeruli with no previous reports related to kidney expression, TRIM6, which has been shown to function as a regulator for Myc-mediated transcription and maintenance of pluripotency in embryonic stem cells [34]. A further three genes - GPR110, SLC25A48 and CRB2 (Fig. 2d) are transmembrane proteins not previously been reported as localised to the glomerulus.

**Figure 3:**
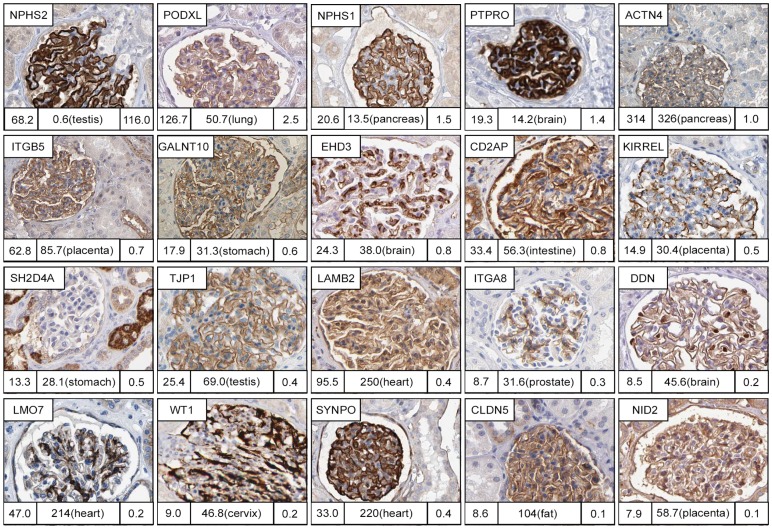
Reverse analysis of proteins previously characterised as glomeruli specific. 4 of 20 glomeruli specific proteins selected based on literature (NPHS2, PODXL, NPHS1 and PTPRO) are found in the group of kidney enriched/enhanced proteins when compared to RNAseq data for the 27 tissues. Furthermore, 6 of the 20 genes do not display a glomeruli-specific localisation within kidney (ITGB5, GALNT10, CD2AP, SH2D4A, LAMB2, CLDN5). Numbers under each IHC image correspond to mean FPKM in kidney (left bottom), Max FPKM in next most highly expressed tissue and the tissue name (middle bottom), and tissue specificity score in kidney (right bottom).

The low number of glomeruli specific proteins that we identified contrasts to the vast literature on the apparent ‘glomeruli specific proteome’ (http://www.hkupp.org/index.htm) [5] [6]. In order to study the expression pattern of these earlier identified glomerus specific proteins, we selected 20 proteins for more in-depth analysis using the immunohistochemistry analysis (Fig. 3) and the genome-wide RNAseq data. Our analysis showed many of the proteins previously identified as glomerulus-specific were also expressed in other tissues, although all the 20 selected proteins were indeed expressed in the glomerus, supporting earlier proteomics studies. Interestingly, although confirmed to be present in the glomerulus (Fig. 3) NEPH1 was not identified as elevated in kidney. Higher mRNA levels were found in the placenta, suggesting an important function in the protein filtration barrier between maternal and fetal circulation, also supported by observations in rat [35] [36]. In all, 16 of the 20 proteins were expressed in another tissue at a higher level and 6 of the 20 proteins were not expressed only in the glomerus (Fig. 3). These results demonstrate the power of comprehensive genome-wide methods for analysis of tissue specificity.

### Highly kidney-enriched genes

**Figure 4:**
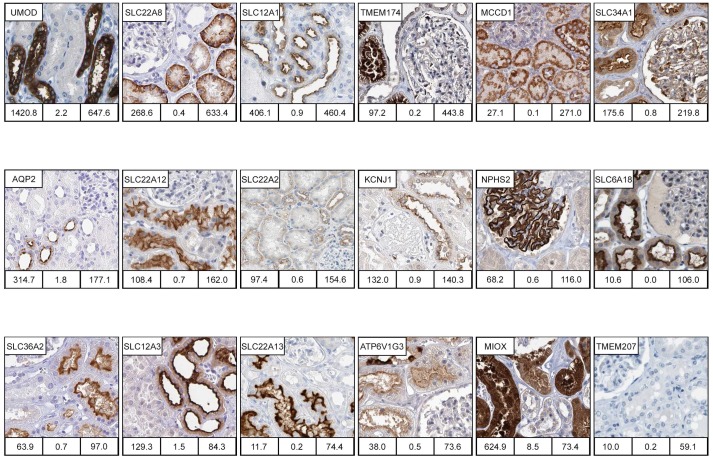
Examples of highly enriched kidney proteins. Immunohistochemically (IHC) stained images of proteins selectively expressed in different subsets of cells and structures in kidney tissue. IHC images were available for 18 of the 20 highly enriched genes, and for 17 of the 18 genes the expression in kidney were confirmed, here ordered according to tissue specificity score in the figure. When ordered according to nephron segment, these are; glomeruli podocytes (NPHS2), proximal tubule cytoplasm (MIOX) and proximal tubule basolateral membrane (SLC22A8, SLC22A2) and proximal tubule luminal membrane (TMEM174, SLC34A1, SLC22A12, SLC6A18, SLC36A2 and SLC22A13), distal tubule cytoplasm (UMOD) and distal tubule luminal membrane (SLC12A1, KCNJ1, SLC12A3), and collecting duct luminal membrane (AQP2). MCCD1 localises the cytoplasm in cells of all tubular segments cytoplasm. ATP6V1G3 localises to luminal membrane of both proximal and distal tubule luminal membrane and with a strong cytoplasmic staining in intercalated cells in collecting duct. TMEM207 could not be confirmed as expressed in kidney in IHC images available in the Protein Atlas. Numbers under each IHC image correspond to mean FPKM in kidney (left bottom), max FPKM of the second most highly expressed tissue (middle bottom), and to Tissue specificity score (TS) in kidney (right bottom).

Of particular interest for kidney biology are the 20 genes identified with highest tissue specific score (Table 1 and S3 File) of which the majority were found to code for transmembrane proteins. Antibody stained tissue sections were available for 18 of the proteins encoded by these genes and 17 showed good concordance between with mRNA and protein expression profiles (Fig. 4). 11 of these genes belong to the family of solute carriers (SLC). Together with the membrane transport proteins KCNJ1 and AQP2, these are involved in specifically regulated processes of transmembrane transport and confined to distinct parts of the tubulus (proximal, distal) and collecting ducts (Fig. 4). A focus on the proximal tubule revealed one protein, SLC22A8, found at the basolateral membrane, while more proteins - SLC34A1, SLC22A12, SLC6A18, SLC36A2 and SLC22A13, were found as localised to the luminal/apical side. In the distal tubule, we found SLC12A1 and SLC12A3 both localised to the luminal/apical side, while SLC22A2 was found both in proximal tubule, localised to the basolateral membrane, and in distal tubule, but here instead localised to luminal/apical membrane (Fig. 4). Two of the genes lacked antibody-based data (SLC22A6 and SLC7A13) and more work is needed to generate antibodies directed towards these solute transporters to identify their nephron-specific expression.

### Enriched genes not previously described in the kidney

**Figure 5:**
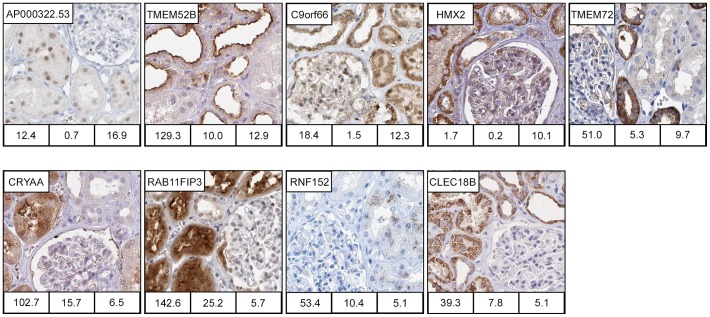
Kidney enriched proteins previously not described in kidney. Immunohisto-chemically stained images of kidney enriched proteins (TS>5 FPKM) for which we found no previous description expression in kidney based searches in literatures or online databases (GeneCards, WikiGene, BioGPS). Of the 16 kidney enriched genes not described previously, IHC images confirming kidney specific localisation were available for 10 genes. In proximal tubule cells, AP000322.53 localise to the nucleus while TMEM52B, RAB11FIP3 and CRYAA show a general cytoplasmic staining, and RNF152 shows a granular cytoplasmic staining. In distal tubule, TMEM72 localises to the basolateral membrane. C9orf66 is found in all nephron segments but with different localisations. In glomeruli, it localise to the nucleus, in proximal tubular cells to nucleus and cytoplasm, while in cells in distal tubule and collecting duct only in cytoplasm. Both HMX2 and CLEC18B localises to the cytoplasm of cells in proximal and distal tubule, and also in the collecting duct, however for CLEC18B only in the intercalated cells. Numbers under each IHC image correspond to Mean FPKM in kidney (left bottom), Max FPKM in the tissue with 2^nd^ most highly expressed level (middle bottom), and Tissue specificity score in kidney (right bottom).

Of the 64 kidney enriched genes (S2 Table), 16 had not been previously described in the context of the kidney. Antibody-based profiling revealed that 10, including a number of uncharacterised proteins for which no previous literature exist (e.g. AP000322.53, TMEM52B, c9orf66, TMEM72, and CLEC18B), were expressed specifically in the nephron (Fig. 5). AP000322.53, a putative transmembrane protein of 139 amino acids, was expressed in the nuclei of a subset of both proximal and distal tubular cells. Interestingly, the HPA data revealed the protein is also expressed in testis (Leydig cells) and in many cancers, although not renal cancer. TMEM52B showed a granular staining pattern, predominantly localised towards the basal membrane in proximal tubule and TMEM72 showed a strong staining in distal tubule cells with a granular cytoplasmic and a basal membrane localisation (Fig. 5). In this group, we also find a putative transcription factor, HMX2, localised to distal tubule (Fig. 5).

### Comparative proteomics studies of the kidney

Many kidney proteomic studies have focused on urine as sample material, however the specific origin of proteins and protein fragments in urine is difficult to determine. Tissue proteomics using methods for proteomic content enrichment can enable identification of the specific proteomes of different functional units, compartments and cells of the kidney. For example, isolating glomeruli followed by 2D SDS-PAGE and LC-MS/MS was successful to enable an in depth characterisation of the glomerular proteome [6], [37]. Beyond glomeruli, the proteomes of kidney compartments are not well characterised. Efforts have been made to isolate tubular structures and cells to define the tubular proteome in animals, however the human tubular and collecting duct proteomes remain to be comprehensively characterised [38], [39]. We therefore compared our data to a recent comprehensive analysis of multiple LC-MS/MS shotgun proteomics data sets available from different studies used to define the non-redundant proteomes of plasma, urine and kidney tissue [40]. We found that 51 of our 148 compartment specific proteins (S4 Table) were not represented neither in the urine, plasma nor kidney tissue proteomes defined by Farrah et al. [40]. The majority of these are transmembrane proteins, and the results may reflect technological challenges in separating and identifying membrane components by traditional LC-MS/MS mass spectrometry based methods [41]. Interestingly, among 352 protein described by Farrah et al. [40] as unique to the urine proteome, several proteins we here identified as specific for the proximal tubuli (SLC3A1, SLC34A1, SLC7A8, SLC13A2, SLC28A1, KL, or collecting duct (AQP2) can be found, suggesting that in the urine proteome these actually represent kidney derived tissue leakage proteins. Furthermore, we compared the 148 compartment specific proteins to publically available omics datasets in the Kidney and Urinary Pathway Knowledge base, KUPKB (http://www.kupkb.org/), to datasets of glomeruli proteins in the Human Kidney and Urinary Proteome Project, HKUPP, (http://www.hkupp.org/), and to datasets in the Urinary Exosome Protein Database (http://dir.nhlbi.nih.gov/papers/lkem/exosome/). The results are shown in S4 Table. Interestingly, for several proteins we identify as specific for only one nephron segment or collecting ducts, there is conflicting previous data (when available) between public repositories KUPKB and HKUPP (S4 Table).

The Human Protein Atlas database contains quantitative annotations of protein expression across the different tissues sections using IHC. These IHC annotations (high’, ‘medium’ or ‘low’) are based on the evaluation of a pathologist and give a quantitative measure specified for different tissue parenchymal cells (e.g. proximal tubuli cells, glomerular cells). The parenchyma specific annotations of protein expression in a tissue section could differ from the quantitative average measure of mRNA transcription that is captured by RNAseq analysis, as the proportion of parenchymal cells relative to other cell types (e.g. fibroblasts, cells of the vasculature) differ between tissues and sections. For comparison, we queried the HPA database of IHC-based annotations of protein expression with search criteria that would correspond to either the ‘Group enriched’ or ‘Kidney enriched’ categories in the transcriptome dataset. This query identified 12 proteins annotated as uniquely found in kidney tissue, of which 6 corresponded to genes found among the 20 ‘highly kidney enriched genes’ in the transcriptome analysis (data not shown). The total overlap between the query-based analysis of the quantitative IHC-annotations in the HPA database and the transcriptome analysis was 35 proteins/genes. These 35 genes were classified as ‘Kidney enriched’ (11 genes), ‘Group enriched’ (14 genes) and ‘Kidney enhanced’ (10 genes) in the transcriptomics analysis. Thus, while IHC analysis provide more specific information of protein expression in parenchyma cells in individual tissues, the combination of RNA seq followed by IHC-based confirmation of protein expression and localisation enabled a more comprehensive identification of kidney enriched genes on genome wide level.

## Discussion

Here, we have comprehensively described the kidney elevated transcriptome and proteome. By comparing the kidney transcriptome against the transcriptomes from 26 other organs, we were able to produce a kidney specific profile of gene expression. To our knowledge, this is the first study utilising RNA-seq in an integrated ‘omics’ approach with supplementary immunohistochemistry data to provide a comprehensive characterisation of protein expression in normal human tissues. We show how this combined approach adds value to the IHC-based information of protein expression in the Human Protein Atlas when aiming to identify kidney enriched genes on a genome wide scale.

Our RNAseq analysis is based on kidney tissue from only four individuals, but the high correlation in mRNA profile between the four samples (S2 Fig.) suggest low individual variation. However, for follow-up studies using the same approach to study the kidney proteome in different pathological conditions, analysis of larger sample numbers is likely to be necessary to accommodate for known disease heterogeneity between individuals.

We validated this profile using immunohistochemical staining, which allowed us to identify specific sub-compartments or cell types where these proteins were expressed (S2 Table and S4 Table). Importantly, this allowed us to identify both novel uncharacterised kidney-specific proteins, as well as known genes that had never previously described in the context of the kidney. The elevated expression reflect active differential transcriptional regulation of genes with a role in kidney that go beyond functions common to many or all tissues and cells and should be of interest for further research aiming to better understand kidney biology.

Furthermore, our results can also provide a resource for more clinically applied research since expression specificity of a protein is useful in the quest for specific markers of injury or disease of a particular tissue or cell type, e.g. elevated plasma levels. From this perspective, our list of identified kidney-enriched proteins with specific compartment location (S4 Table) provide interesting candidates for more in-depth studies in plasma or urine.

To conclude, we here provide a comprehensive knowledge resource identifying the kidney transcriptome and, importantly, the specific localisation of expression of the corresponding proteins. We envisage that this will be a useful tool for researchers working in both basic and clinical nephrology research.

## Supporting Information

S1 Fig **Histology of the samples used for RNA-Seq.** Hematoxylin-eosin stained frozen sections of kidney tissue, showing the histology of the four individual kidney samples used for RNA-Seq preparation. All four samples display histologically normal kidney tissue. Numbers indicate the percentage of glomeruli, tubule, endothelial, fibroblasts, smooth muscle and inflammatory cells in each biopsy section chosen for RNA seq.
(TIF)

S2 Fig **Correlation of genes expressed in kidney in relation to gene expression in other tissues.** (A) The correlation between two kidney samples from different individuals shown as a scatterplot of FPKM values for all detected genes. The Spearman pairwise correlations across all genes ranged from 0.96 to 0.98 between different kidney samples. (B) Scatter plot of average FPKM values for all detected genes in four kidney samples (x-axis) and seven testis samples (y-axis) from different individuals (pairwise Spearman correlation coefficient of 0.69).
(TIF)

S1 Table **Top 30 expressed genes in kidney (based on FPKM).**
(PDF)

S2 Table **All kidney enriched genes.**
(PDF)

S3 Table **The 39 shared proteins between kidney and liver.**
(PDF)

S4 Table **The nephron segment and collecting duct specific proteins identified in this study.**
(PDF)

S5 Table **GO analyses of the different nephron segment and collecting duct specific proteins.**
(PDF)

S6 Table **GO analyses of the group enriched proteins.**
(PDF)

S1 File **Kidney enhanced genes.**
(XLS)

S2 File **Group enriched genes.**
(XLS)

S3 File **The highly kidney enriched genes of** **Table 1** **with measures of variance and individual FPKM values of four individual kidney samples.**
(XLS)
