# Supplementary figures and images for: The Kidney Transcriptome and Proteome Defined by Transcriptomics and Antibody-Based Profiling
Source: PLoS One. 2014 Dec 31;9(12):e116125. doi: 10.1371/journal.pone.0116125 (PMC4281243; doi:10.1371/journal.pone.0116125)

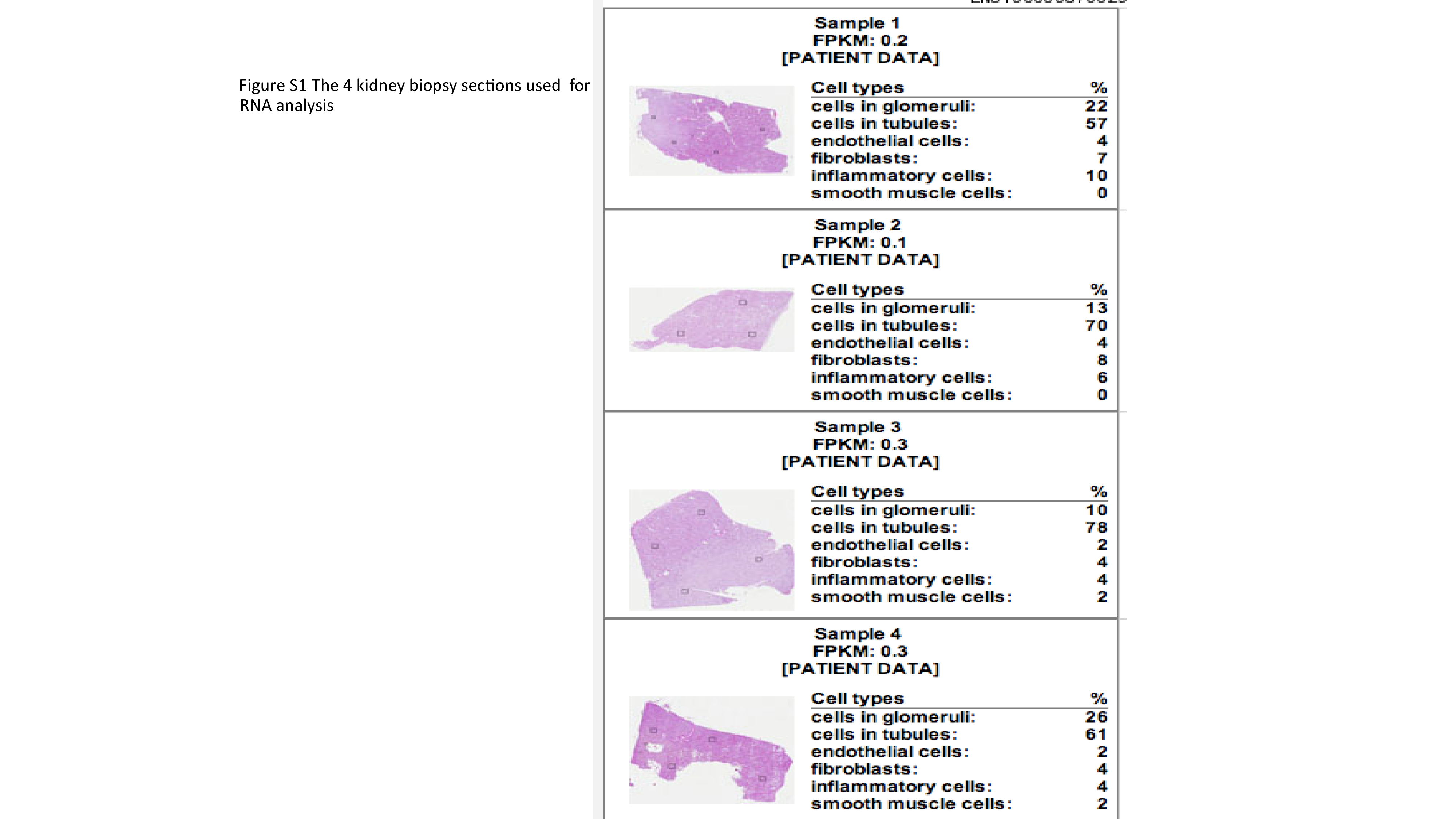

Supplement: S1 Fig — Histology of the samples used for RNA-Seq. Hematoxylin-eosin stained frozen sections of kidney tissue, showing the histology of the four individual kidney samples used for RNA-Seq preparation. All four samples display histologically normal kidney tissue. Numbers indicate the percentage of glomeruli, tubule, endothelial, fibroblasts, smooth muscle and inflammatory cells in each biopsy section chosen for RNA seq. (TIF) [file pone.0116125.s001.tif]

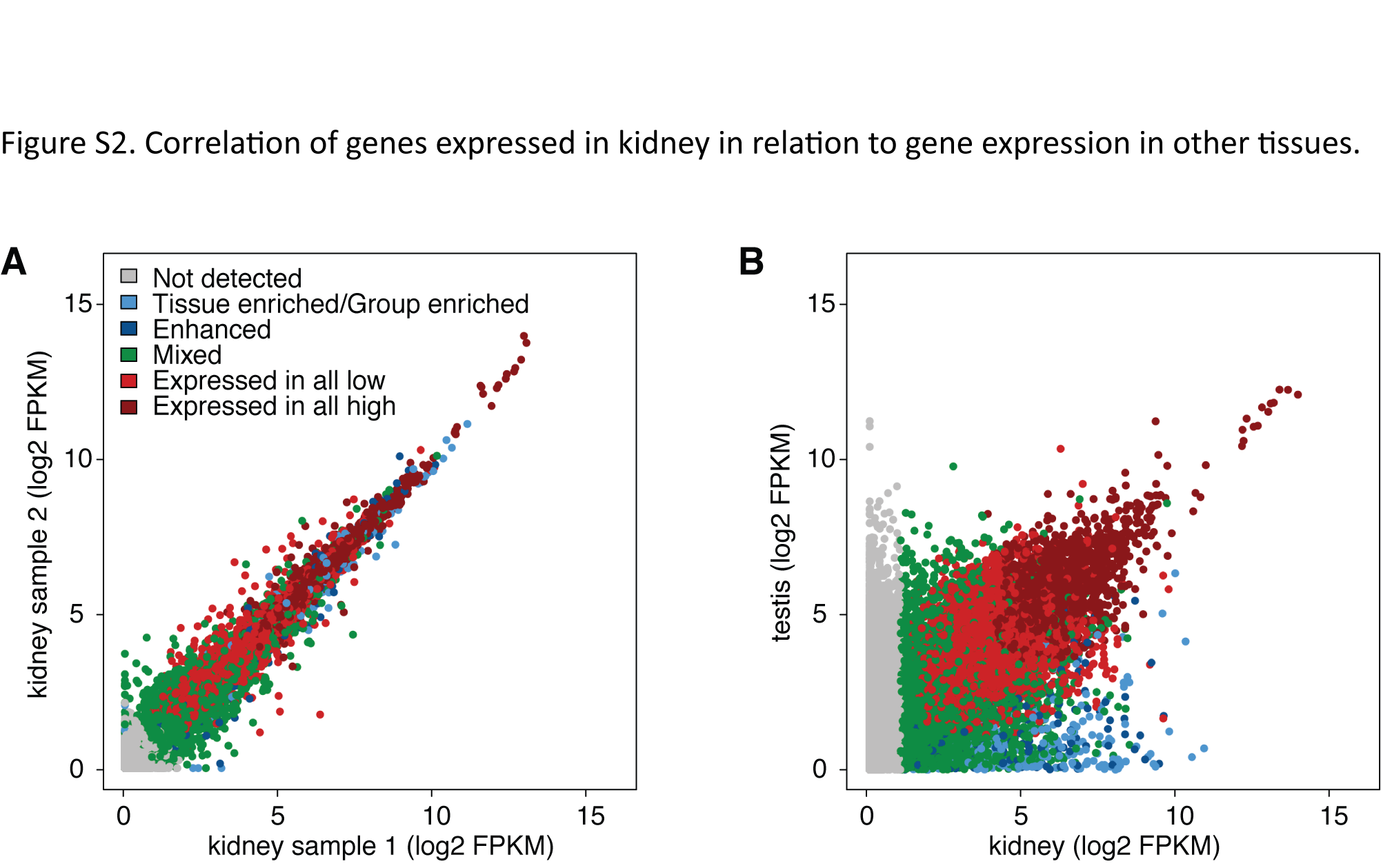

Supplement: S2 Fig — Correlation of genes expressed in kidney in relation to gene expression in other tissues. (A) The correlation between two kidney samples from different individuals shown as a scatterplot of FPKM values for all detected genes. The Spearman pairwise correlations across all genes ranged from 0.96 to 0.98 between different kidney samples. (B) Scatter plot of average FPKM values for all detected genes in four kidney samples (x-axis) and seven testis samples (y-axis) from different individuals (pairwise Spearman correlation coefficient of 0.69). (TIF) [file pone.0116125.s002.tif]
