# Supplementary material for: The Kidney Transcriptome and Proteome Defined by Transcriptomics and Antibody-Based Profiling
Source: PLoS One. 2014 Dec 31;9(12):e116125. doi: 10.1371/journal.pone.0116125 (PMC4281243; doi:10.1371/journal.pone.0116125)
Supplement: S1 Table — Top 30 expressed genes in kidney (based on FPKM). (PDF) [file pone.0116125.s003.pdf]

Table S1. The top 30 expressed genes in kidney, ranked by FPKM values

Gene_name	Description	SP/TM-category	Category	Proteinclasses	TS-score	Max fpkm	Max other	Mean fpkm	Kidney fpkm	Kidney enhanced score
J01415.25	ATP synthase protein 8	TM	Expressed in all high		1,3	24076,8	18658,6	7896,0	15484,5	2,0
MT-CO1	mitochondrially encoded cytochrome c oxidase I	TM	Expressed in all high	Transporters,Enzymes	1,5	19182,9	12381,1	6779,9	12381,1	1,8
MT-CO3	mitochondrially encoded cytochrome c oxidase III	TM	Expressed in all high	Transporters	1,5	14940,2	10224,0	5752,4	10224,0	1,8
MT-ATP6	mitochondrially encoded ATP synthase 6	TM	Expressed in all high		1,9	17009,0	8976,6	5300,3	8976,6	1,7
MT-ND4	mitochondrially encoded NADH dehydrogenase 4	TM	Expressed in all high	Enzymes	1,5	14034,1	9511,3	4708,6	8365,8	1,8
MT-ND2	mitochondrially encoded NADH dehydrogenase 2	TM	Expressed in all high		1,0	11515,4	11002,7	3994,9	7938,9	2,0
MT-CO2	mitochondrially encoded cytochrome c oxidase II	TM	Expressed in all high	Transporters	1,5	11069,0	7276,6	4439,5	6884,1	1,6
MT-CYB	mitochondrially encoded cytochrome b	TM	Expressed in all high		1,4	8772,8	6266,4	3143,1	6266,4	2,0
MT-ND4L	mitochondrially encoded NADH dehydrogenase 4L	TM	Expressed in all high	Enzymes	1,3	7460,8	5703,4	2906,4	5703,4	2,0
MT-ND1	mitochondrially encoded NADH dehydrogenase 1	TM	Expressed in all high	Enzymes	1,4	8035,4	5561,5	2767,3	4871,4	1,8
MT-ND6	mitochondrially encoded NADH dehydrogenase 6	TM	Expressed in all high	Enzymes	1,7	8003,5	4756,7	2382,6	4544,7	1,9
MT-ND3	mitochondrially encoded NADH dehydrogenase 3	TM	Expressed in all high	Enzymes	1,6	8588,5	5236,3	3088,8	4440,1	1,4
MT-ND5	mitochondrially encoded NADH dehydrogenase 5	TM	Expressed in all high	Enzymes	1,7	7358,3	4389,9	2007,2	4389,9	2,2
B2M	beta-2-microglobulin	INTRA-SP	Expressed in all high	Plasma proteins	1,0	5523,5	5438,9	3359,2	1941,4	0,6
ALDOB	aldolase B, fructose-bisphosphate	INTRA	Group enriched	Enzymes	1,0	1935,4	1867,0	270,2	1867,0	6,9
EEF1A1	eukaryotic translation elongation factor 1 alpha 1	INTRA	Expressed in all high		1,1	1990,8	1834,1	1201,7	1722,5	1,4
TPT1	tumor protein, translationally-controlled 1	INTRA	Expressed in all high		1,2	2701,9	2301,8	1553,6	1539,0	1,0
FTL	ferritin, light polypeptide	INTRA	Expressed in all high	Plasma proteins	1,1	1593,9	1488,8	860,8	1461,8	1,7
UMOD	uromodulin	INTRA-SP-TM	Tissue specific		647,6	1420,8	2,2	53,0	1420,8	26,8
SPP1	secreted phosphoprotein 1	SP	Group enriched		1,1	1794,3	1669,6	206,4	1230,9	6,0
GATM	glycine amidinotransferase (L-arginine:glycine amidinotransferase)	INTRA	Mixed High	Enzymes	1,2	1127,9	974,2	151,2	974,2	6,4
ITM2B	integral membrane protein 2B	TM	Expressed in all high		1,3	900,7	687,6	428,9	900,7	2,1
FXYD2	FXYD domain containing ion transport regulator 2	TM	Group enriched	Transporters	1,5	851,4	559,9	76,6	851,4	11,1
ATP1B1	ATPase, Na+/K+ transporting, beta 1 polypeptide	INTRA-TM	Expressed in all low	Transporters	2,4	840,5	349,7	162,2	840,5	5,2
LDHB	lactate dehydrogenase B	INTRA	Expressed in all high	Plasma proteins,Enzymes	1,4	1129,6	822,2	263,4	822,2	3,1
UBB	ubiquitin B	INTRA	Expressed in all high		1,0	899,4	893,5	626,3	815,4	1,3
GSTA1	glutathione S-transferase alpha 1	INTRA	Mixed high	Enzymes	1,4	1135,2	810,1	184,6	810,1	4,4
RPL41	ribosomal protein L41	INTRA	Expressed in all high	Ribosomal proteins	1,7	2617,5	1582,3	1114,8	783,3	0,7
PDZK1IP1	PDZK1 interacting protein 1	TM	Mixed high		4,0	751,5	187,9	59,1	751,5	12,7
MT1G	metallothionein 1G	INTRA-SP	Expressed in all low		1,3	1001,5	744,3	163,8	744,3	4,5
