# Supplementary material for: The Kidney Transcriptome and Proteome Defined by Transcriptomics and Antibody-Based Profiling
Source: PLoS One. 2014 Dec 31;9(12):e116125. doi: 10.1371/journal.pone.0116125 (PMC4281243; doi:10.1371/journal.pone.0116125)
Supplement: S2 Table — All kidney enriched genes. (PDF) [file pone.0116125.s004.pdf]

Table S2. The kidney enriched genes

Gene name	Description	Localization	Cellular Compartment	Kidney mRNA	TS score	q-value
UMOD	Uromodulin	Distal tubule	Membrane	1421	647,6	2,80E-035
SLC22A8	Solute carrier family 22, member 8	Proximal tubule	Membrane	269	633,4	2,60E-032
SLC12A1	Solute carrier family 12, member 1	Distal tubule	Membrane	406	460,4	2,90E-027
TMEM174	Transmembrane protein 174	Proximal tubule	Membrane	97	443,8	2,90E-027
MCCD1	Mitochondrial coiled-coil domain 1	Tubule	Membrane	27	271,0	1,10E-026
SLC34A1	Solute carrier family 34, member 1	Proximal tubule	Membrane	176	219,8	3,00E-028
AQP2	Aquaporin 2 (collecting duct)	Collecting duct	Membrane	315	177,1	8,10E-022
SLC22A12	Solute carrier family 22, member 12	Proximal tubule	Membrane	108	162,0	4,10E-032
SLC22A2	Solute carrier family 22, member 2	Tubule	Membrane	97	154,6	1,10E-023
SLC22A6	Solute carrier family 22, member 6	No IHC	Membrane	198	144,5	7,50E-027
KCNJ1	Potassium inwardly-rectifying channel	Distal tubule	Membrane	132	140,3	3,10E-023
SLC7A13	Solute carrier family 7, membrane 13	No IHC	Membrane	19	118,7	2,10E-014
NPHS2	Nephrosis 2, idiopathic, (podocin)	Glomeruli	Membrane	68	116,0	2,10E-020
SLC6A18	Solute carrier family 6, member 18	Proximal tubule	Membrane	11	106,0	2,00E-015
SLC36A2	Solute carrier family 36, member 2	Proximal tubule	Membrane	64	97,0	1,50E-017
SLC12A3	Solute carrier family 12, member 3	Tubule	Membrane	129	84,3	4,20E-023
SLC22A13	Solute carrier family 22, member 13	Proximal tubule	Membrane	12	74,4	6,90E-015
ATP6V1G3	ATPase, H+ transporting, lysosomal	Tubule	Membrane	38	73,6	1,60E-021
MIOX	Myo-inositol oxygenase	Proximal tubule	Cytoplasm	625	73,4	8,10E-022
TMEM207	Transmembrane protein 207	Negative	Membrane	10	59,1	1,10E-017
LHX1	LIM homeobox 1	Glomeruli and tubule	Nuclear	8	46,7	2,50E-018
SLC5A10	Solute carrier family 5, member 10	Tubule	Membrane	48	27,5	1,50E-009
SLC22A24	Solute carrier family 22, member 24	Glomeruli and tubule	Membrane	3	25,9	1,10E-018
CTXN3	Cortexin 3	No IHC	Membrane	44	23,0	2,50E-015
SLC4A9	Solute carrier family 4, member 9	Collecting duct	Membrane	11	18,8	8,50E-012
CALB1	Calbindin 1, 28kDa	Distal tubule	Cytoplasm	230	18,7	3,90E-017
SOST	Sclerostin	Tubule	Secreted	11	17,8	2,00E-015
ATP6V0D2	ATPase, H+ transporting, lysosomal	Distal tubule	Cytoplasm	41	17,8	3,80E-009
AP000322.53	No name	Tubule	Nuclear	12	16,9	2,30E-006
FXYD4	FXYD domain ion transport regulator 4	Interstitium	Membrane	80	15,9	1,30E-006
NOX4	NADPH oxidase 4	Proximal tubule	Membrane	114	15,7	6,60E-011
NAT8	N-acetyltransferase 8	Proximal tubule	Membrane	528	13,4	2,80E-018
TMEM52B	Transmembrane protein 52B	Proximal tubule	Membrane	129	12,9	4,30E-012
SLC6A13	Solute carrier family 6, member 13	Distal tubule	Membrane	141	12,6	1,20E-011
SLC47A2	Solute carrier family 47, member 2	Glomeruli and tubule	Membrane	48	12,4	2,90E-011
C9orf66	Chromosome 9 open reading frame 66	Tubule	Cytoplasm	18	12,3	4,00E-005
AQP6	Aquaporin 6, kidney specific	Tubule	Membrane	17	11,3	5,30E-007
HMX2	H6 family homeobox 2	Tubule	Cytoplasm	2	10,1	1,00E-007
TMEM72	Transmembrane protein 72	Distal tubule	Membrane	51	9,7	5,50E-008
C16orf11	Chromosome 16 open reading frame 11	No IHC	Cytoplasm	4	9,5	9,40E-008
PAX2	Paired box 2	Proximal tubule	Nuclear	36	9,2	1,60E-015
TMEM27	TransMembraneprotein 27	Tubule	Membrane	219	9,0	5,50E-012
UPP2	Uridine phosphorylase 2	Glomeruli and tubule	Cytoplasm	28	8,8	2,90E-010
PVALB	Parvalbumin	Distal tubule	Cytoplasm	40	8,4	1,30E-006
PTH1R	Parathyroid hormone 1 receptor	Distal tubule	Membrane	183	8,4	6,40E-006
OR2T10	Olfactory receptor 2, member 10	Negative	Membrane	4	7,9	0,00094
CDH16	Cadherin 16, KSP-cadherin	Distal tubule	Membrane	226	7,5	2,60E-011
SLC5A2	Solute carrier family 5, member 2	Proximal tubule	Membrane	50	7,1	4,40E-008
FMO1	Flavin containing monooxygenase 1	Proximal tubule	Membrane	185	7,0	1,70E-006
CRYAA	Crystallin, alpha A	Proximal tubule	Cytoplasm	103	6,5	1,80E-012
CYS1	Cystin 1	No IHC	Cytoplasm	99	6,5	4,00E-005
SIM1	Single-minded homolog 1 (Drosophila)	Distal tubule	Cytoplasm	11	6,4	3,80E-010
SLC13A3	Solute carrier family 13, member 3	Glomeruli and tubule	Membrane	305	6,3	7,40E-010
EMX1	Empty spiracles homeobox 1	Distal tubule	Cytoplasm	28	6,2	3,50E-007
CLCNKA	Chloride channel, voltage-sensitive Ka	Distal tubule	Membrane	62	6,0	0,0099
RAB11FIP3	RAB11 family interacting protein 3	Proximal tubule	Cytoplasm	143	5,9	0,9300
TINAG	Tubulointerstitial nephritis antigen	Tubule	Cytoplasm	78	5,8	4,46E-008
GALNT14	Acetylgalactosaminyltransferase 14	Proximal tubule	Membrane	56	5,7	0,0016
TRPV5	Transient receptor, member 5	Glomeruli and tubule	Membrane	1	5,6	0,0049
SLC16A12	Solute carrier family 16, member 12	Proximal tubule	Membrane	62	5,4	1,82E-005
C10orf126	Chromosome 10 frame 126	Proximal tubule	Nuclear	2	5,4	1,51E-005
A2LD1	AIG2-like domain 1	No IHC	Cytoplasm	63	5,3	0,0026
RNF152	Ring finger protein 152	Proximal tubule	Membrane	53	5,1	0,0160
CLEC18B	C-type lectin family 18, member B	Tubule	Secreted	39	5,1	0,00019
