# Supplementary material for: The Kidney Transcriptome and Proteome Defined by Transcriptomics and Antibody-Based Profiling
Source: PLoS One. 2014 Dec 31;9(12):e116125. doi: 10.1371/journal.pone.0116125 (PMC4281243; doi:10.1371/journal.pone.0116125)
Supplement: S3 Table — The 39 shared proteins between kidney and liver. (PDF) [file pone.0116125.s005.pdf]

Table S3. The 39 kidney/liver shared proteins

Gene_name	Description	Proteinclasses	group tissues	Group-specific score	Kidney fpkm	Liver fpkm
CLCN5	chloride channel, voltage-sensitive 5		kidney,liver	5.1	41.1	8.7
SLC17A3	solute carrier family 17 (sodium phosphate), member 3	Transporters	kidney,liver	125.6	101.7	23.1
SLC17A1	solute carrier family 17 (sodium phosphate), member 1	Transporters	kidney,liver	35.3	97.4	24.7
UGT1A9	UDP glucuronosyltransferase 1 family, polypeptide A9	Enzymes	kidney,liver	191.3	252.9	65.0
PRODH2	proline dehydrogenase (oxidase) 2	Enzymes	kidney,liver	72.5	201.4	67.9
AGXT2	alanine--glyoxylate aminotransferase 2	Enzymes	kidney,liver	21.3	114.0	52.1
ACSM2A	acyl-CoA synthetase medium-chain family member 2A	Enzymes	kidney,liver	386.2	281.6	193.4
BHMT	betaine--homocysteine S-methyltransferase	Enzymes	kidney,liver	127.3	559.7	401.4
ACOT6	acyl-CoA thioesterase 6		kidney,liver	5.6	1.6	1.2
BHMT2	betaine--homocysteine S-methyltransferase 2	Enzymes	kidney,liver	7.6	331.9	255.0
ACMSD	aminocarboxymuconate semialdehyde decarboxylase	Enzymes	kidney,liver	11.1	85.9	66.8
AFP	alpha-fetoprotein	Plasma proteins	kidney,liver	55.9	16.1	12.9
UGT2B7	UDP glucuronosyltransferase 2 family, polypeptide B7	Enzymes	kidney,liver	8.5	597.5	485.3
HAO2	hydroxyacid oxidase 2 (long chain)	Enzymes	kidney,liver	447.9	198.3	172.6
DMGDH	dimethylglycine dehydrogenase	Enzymes	kidney,liver	10.6	130.0	116.0
ACSM2B	acyl-CoA synthetase medium-chain family member 2B	Enzymes	kidney,liver	264.1	319.5	293.3
DAO	D-amino-acid oxidase	Enzymes	kidney,liver	47.0	58.8	56.9
ASS1	argininosuccinate synthase 1	Enzymes	kidney,liver	5.1	730.4	793.9
UGT2A1	UDP glucuronosyltransferase 2 family, polypeptide A1, complex locus	Enzymes	kidney,liver	19.5	14.9	16.9
SLC27A2	solute carrier family 27 (fatty acid transporter), member 2	Transporters,Enzymes	kidney,liver	5.5	93.3	115.9
ASPDH	aspartate dehydrogenase domain containing	Enzymes	kidney,liver	6.7	30.2	37.9
DPYS	dihydropyrimidinase	Enzymes	kidney,liver	15.3	177.5	231.4
RIPPLY1	ripply1 homolog (zebrafish)		kidney,liver	9.1	2.5	3.5
DNMT3L	DNA (cytosine-5-)-methyltransferase 3-like		kidney,liver	17.5	4.1	5.9
EHHADH	enoyl-CoA, hydratase/3-hydroxyacyl CoA dehydrogenase	Enzymes	kidney,liver	7.5	116.5	167.4
GLYATL1	glycine-N-acyltransferase-like 1	Enzymes	kidney,liver	12.8	239.9	364.1
PROZ	protein Z, vitamin K-dependent plasma glycoprotein	Peptidases,Plasma proteins	kidney,liver	28.6	15.4	25.3
CLDN14	claudin 14		kidney,liver	13.3	5.3	9.0
ALDH8A1	aldehyde dehydrogenase 8 family, member A1		kidney,liver	120.5	70.7	123.5
FTCD	formiminotransferase cyclodeaminase	Enzymes	kidney,liver	9.2	148.1	260.3
APOM	apolipoprotein M		kidney,liver	15.3	109.8	218.9
HRSP12	heat-responsive protein 12		kidney,liver	8.9	297.7	596.5
PIPOX	pipecolic acid oxidase	Enzymes	kidney,liver	13.9	105.1	213.5
CYP4A11	cytochrome P450, family 4, subfamily A, polypeptide 11	Enzymes	kidney,liver	49.4	197.2	460.7
HPD	4-hydroxyphenylpyruvate dioxygenase	Enzymes	kidney,liver	109.7	306.1	878.7
SLC22A7	solute carrier family 22 (organic anion transporter), member 7	Transporters	kidney,liver	65.0	47.4	141.6
UPB1	ureidopropionase, beta	Enzymes	kidney,liver	22.7	39.4	120.6
CYP4A22	cytochrome P450, family 4, subfamily A, polypeptide 22	Enzymes	kidney,liver	494.6	33.2	120.2
KNG1	kininogen 1	Plasma proteins	kidney,liver	344.9	271.2	1035.8
