# Supplementary material for: The Kidney Transcriptome and Proteome Defined by Transcriptomics and Antibody-Based Profiling
Source: PLoS One. 2014 Dec 31;9(12):e116125. doi: 10.1371/journal.pone.0116125 (PMC4281243; doi:10.1371/journal.pone.0116125)
Supplement: S4 Table — The nephron segment and collecting duct specific proteins identified in this study. (PDF) [file pone.0116125.s006.pdf]

Table S4. Nephron segment-specific localized genes with kidney specificity.

Gene name	Description	Category RNA	FPKM	Public database (mRNA level)		Public database (protein level)		Public database (Urine proteome)	Farrah (1)	Literature
				Database	Nephron segment	Database	Nephron segment			
**a. Glomeruli elevated genes**										
PODXL	podocalyxin-like	Enhanced	126,7	KUPKB	g	KUPKB	g	KUPKB, UEPD	k/u/pl	k, g (2)
TSPAN33	tetraspanin 33	Enhanced	98,3	KUPKB	p					
FGF1	fibroblast growth factor 1 (acidic)	Enhanced	68,5	KUPKB	g,p	KUPKB	g		k	k, g, d (3)
NPHS2	nephrosis 2, idiopathic, (podocin)	Highly enriched	68,2	KUPKB	g	KUPKB	g	UEPD	k/u	k, g (4)
SLC5A3	solute carrier family 5, member 3	Enhanced	28,2	KUPKB	k (n.s)					k, g (5)
PTH2R	parathyroid hormone 2 receptor	Enhanced	23,3							k, g (6)
GPR110	G protein-coupled receptor 110	Enhanced	22,8							k, (7)
TRIM6	tripartite motif containing 6	Enhanced	20,8	KUPKB	p					
NPHS1	nephrosis 1, congenital, Finnish type (nephrin)	Group enriched	20,6	KUPKB	g	KUPKB,HKUPP	g	KUPKB, UEPD	k	k, g (8)
PTPRO	protein tyrosine phosphatase, receptor type, O	Enhanced	19,3	KUPKB	g	KUPKB,HKUPP	g	KUPKB, UEPD	k/u	k, g (9)
SLC25A48	solute carrier family 25, member 48	Enhanced	12,8	KUPKB	p,d,c					
CRB2	crumbs homolog 2 (Drosophila)	Enhanced	5,1	KUPKB	p	KUPKB,HKUPP	g	KUPKB, UEPD	k/u/pl	k, (10)
**b. Proximal tubuli elevated genes**										
ALDOB	aldolase B, fructose-bisphosphate	Group enriched	1867	KUPKB	p,d,c	KUPKB, HKUPP	g	KUPKB	k/u/pl	k, (11)
GATM	glycine amidinotransferase	Group enriched	974,2	KUPKB	p,d,c	KUPKB, HKUPP	g	KUPKB	k/u/pl	k, p (12)
PDZK1IP1	PDZK1 interacting protein 1	Enhanced	751,5	KUPKB	g,p,d,c	KUPKB, HKUPP	g	KUPKB, UEPD	k/u	k, p (13)
ASS1	argininosuccinate synthase 1	Group enriched	730,4	KUPKB	g,p,d,c	KUPKB, HKUPP	g	KUPKB, UEPD	k/u/pl	k, p (14)
MIOX	myo-inositol oxygenase	Highly enriched	624,9	KUPKB	p,d,c				k/u	k, p (15)
PAH	phenylalanine hydroxylase	Enhanced	570,5	KUPKB	g	KUPKB	k			k, p (16)
BHMT	betaine--homocysteine S-methyltransferase	Group enriched	559,7	KUPKB	g,p,d,c	KUPKB, HKUPP	g	KUPKB	k/u/pl	k, p (17)
NAT8	N-acetyltransferase 8 (GCN5-related, putative)	Moderately enriched	527,5	KUPKB	p,d,c					k, (18)
PCK1	phosphoenolpyruvate carboxykinase 1 (soluble)	Enhanced	493,6	KUPKB	p,d,c	KUPKB, HKUPP	g	KUPKB, UEPD	k/u	k, p (19)
C19orf77	chromosome 19 open reading frame 77	Enhanced	442,6	KUPKB	p			KUPKB, UEPD	k/u	
GSTA2	glutathione S-transferase alpha 2	Enhanced	393,7	KUPKB	k (n.s)	KUPKB, HKUPP	g	KUPKB	k/u/pl	k, p (20)
PDZK1	PDZ domain containing 1	Enhanced	390,4	KUPKB	p,d,c	KUPKB, HKUPP	g	KUPKB, UEPD	k/u/pl	k, p (21)
ACY1	aminoacylase 1	Group enriched	346,8	KUPKB	p,d,c	KUPKB, HKUPP	g	KUPKB, UEPD	k/u/pl	k, p (22)
BHMT2	betaine--homocysteine S-methyltransferase 2	Group enriched	331,9			KUPKB	k	KUPKB, UEPD	k/u/pl	k, (23)
ACSM2B	acyl-CoA synthetase medium-chain member 2B	Group enriched	319,5			KUPKB	g		k/u	
PEPD	peptidase D	Enhanced	314,8	KUPKB	p,d,c	KUPKB, HKUPP	g	KUPKB, UEPD	k/u/pl	
SLC3A1	solute carrier family 3, member 1	Enhanced	314,1	KUPKB	p,d,c			UEPD	u	k, p (24)
HPD	4-hydroxyphenylpyruvate dioxygenase	Group enriched	306,1	KUPKB	p			KUPKB, UEPD	k/u/pl	k, (25)
SLC13A3	solute carrier family 13, member 3	Moderately enriched	304,5	KUPKB	p, d	KUPKB, HKUPP	g	UEPD		k, p (26)
HRSP12	heat-responsive protein 12	Group enriched	297,7	KUPKB	g,p	KUPKB, HKUPP	g	KUPKB, UEPD	k/u/pl	k, (27)
ACSM2A	acyl-CoA synthetase medium-chain member 2A	Group enriched	281,6			KUPKB, HKUPP	g		k/u	

**Proximal tubuli elevated genes (continued)**										
KNG1	kininogen 1	Group enriched	271,2	KUPKB	p, d	KUPKB, HKUPP	g	KUPKB, UEPD	k/u/pl	k, (28)
SLC22A8	solute carrier family 22, member 8	Highly enriched	268,6	KUPKB	p, d			UEPD	k	k, p (29)
GLYAT	glycine-N-acyltransferase	Group enriched	268	KUPKB	p	KUPKB, HKUPP	g		k	k, (30)
C11orf54	chromosome 11 open reading frame 54	Enhanced	265,4	KUPKB	p,d,c	KUPKB, HKUPP	g	UEPD	k/u/pl	k, (31)
BBOX1	butyrobetaine2-oxoglutarate dioxygenase 1	Enhanced	255,6	KUPKB	p,d,c			UEPD	k/u	k, (32)
DPEP1	dipeptidase 1 (renal)	Enhanced	249,4	KUPKB	p,d,c	KUPKB, HKUPP	g	KUPKB, UEPD	k/u/pl	k, p (33)
CMBL	carboxymethylenebutenolidase	Enhanced	246,8	KUPKB	p,d,c	KUPKB, HKUPP	g	UEPD	k/u/pl	k, (34)
GLYATL1	glycine-N-acyltransferase-like 1	Group enriched	239,9			KUPKB, HKUPP	g		k	k, (35)
DDC	dopa decarboxylase	Enhanced	223,9	KUPKB	p	KUPKB, HKUPP	g	KUPKB, UEPD	k/u/pl	k, p (36)
KHK	ketohexokinase (fructokinase)	Group enriched	208	KUPKB	p, d	KUPKB, HKUPP	g	UEPD	k/u/pl	k, p (37)
SLC7A7	solute carrier family 7, member 7	Enhanced	201,7	KUPKB	k (n.s)					k, (38)
FMO1	flavin containing monooxygenase 1	Moderately enriched	184,8	KUPKB	p				k	k, p (39)
SHMT1	serine hydroxymethyltransferase 1 (soluble)	Enhanced	184,7	KUPKB	p,d,c	KUPKB	k	KUPKB, UEPD	k/u/pl	k, p (40)
DHRS4	dehydrogenase/reductase member 4	Enhanced	184	KUPKB	p,d,c	KUPKB, HKUPP	g		k	
DPYS	dihydropyrimidinase	Group enriched	177,5	KUPKB	p, d	HKUPP	g	KUPKB, UEPD	k/u	k, (41)
PBLD	phenazine biosynthesis-like protein domain	Enhanced	177,1	KUPKB	g,p,d,c	KUPKB, HKUPP	g	KUPKB, UEPD	k/u/pl	
SLC34A1	solute carrier family 34, member 1	Highly enriched	175,6	KUPKB	g,p, d				u	k, p (42)
PCK2	Phosphoenolpyruvate carboxykinase 2	Group enriched	169	KUPKB	p,d,c	KUPKB, HKUPP	g		k/u/pl	k, (43)
FAM151A	family with sequence similarity 151, member A	Group enriched	168,2					KUPKB	k/u	
SLC7A8	solute carrier family 7, member 8	Enhanced	158,7						u	k, (44)
FTCD	formiminotransferase cyclodeaminase	Group enriched	148,1	KUPKB	p,c	KUPKB, HKUPP	g	UEPD	k/u	k, (45)
XPNPEP2	X-prolyl aminopeptidase (aminopeptidase P) 2,	Group enriched	134,3	KUPKB				KUPKB, UEPD	k/u/pl	
AGMAT	agmatine ureohydrolase (agmatinase)	Enhanced	130,2			KUPKB, HKUPP	g		k/pl	k, (46)
DMGDH	dimethylglycine dehydrogenase	Group enriched	130			KUPKB, HKUPP	g		k/pl	k, p (47)
AK4	adenylate kinase 4	Enhanced	127,9	KUPKB	k (n.s)	KUPKB, HKUPP	g		k/u/pl	k, p (48)
GGT1	gamma-glutamyltransferase 1	Enhanced	126,9	KUPKB	p,d,c	KUPKB, HKUPP	g	KUPKB	k/u/pl	k, p (49)
KL	klotho	Group enriched	120,6	KUPKB	p			UEPD	u	k, p (50)
CUBN	cubilin (intrinsic factor-cobalamin receptor)	Group enriched	120,6	KUPKB	k (n.s)	KUPKB, HKUPP	g	KUPKB, UEPD	k/u/pl	k, p (51)
EHHADH	enoyl-CoA,,hydrataselCoA dehydrogenase	Group enriched	116,5	KUPKB	g,p, d	KUPKB, HKUPP	g		k	k, p (52)
AGXT2	alanine--glyoxylate aminotransferase 2	Group enriched	114			KUPKB	k			k, (53)
NOX4	NADPH oxidase 4	Moderately enriched	113,8			KUPKB, HKUPP	g			k, d (54)
APOM	apolipoprotein M	Group enriched	109,8	KUPKB	p, c				k/u/pl	k, p (55)
SLC16A4	solute carrier family 16, member 4	Enhanced	109,4	KUPKB	p,d,c					k, p (56)
SLC22A12	solute carrier family 22, member 12	Highly enriched	108,4					UEPD	k/u	k, p (57)
QPRT	quinolinate phosphoribosyltransferase	Enhanced	108,2	KUPKB	g,p,d,c			KUPKB	k/u/pl	k, (58)
SLC16A9	solute carrier family 16, member 9	Enhanced	106,4							k, (59)
SLC4A4	solute carrier family 4, , member 4	Enhanced	104	KUPKB	p,d,c			UEPD		k, p (60)
SORD	sorbitol dehydrogenase	Group enriched	103,8	KUPKB	p			KUPKB, UEPD	k/u/pl	k, (61)
CRYAA	crystallin, alpha A	Moderately enriched	102,7	KUPKB	k (n.s)			KUPKB	k	
SLC17A3	solute carrier family 17, member 3	Group enriched	101,7	KUPKB	p,d,c					k, p (62)
TMEM174	transmembrane protein 174	Highly enriched	97,2							k, (63)
SLC6A19	solute carrier family 6, member 19	Group enriched	94,9					KUPKB, UEPD	k/u	k, p (64)
SLC27A2	solute carrier family 27, member 2	Group enriched	93,3	KUPKB	p	KUPKB	k	UEPD	k/u	k, (65)
ACMSD	Aminocarboxymuconate decarboxylase	Group enriched	85,9	KUPKB	p					k, (66)
BPHL	biphenyl hydrolase-like (serine hydrolase)	Enhanced	79,9	KUPKB	k (n.s)	KUPKB, HKUPP	g		k	k, (67)

**Proximal tubuli elevated genes (continued)**										
LRP2	low density lipoprotein receptor protein 2	Group enriched	74,4	KUPKB	k (n.s)	KUPKB, HKUPP	g	KUPKB, UEPD	k/u	k, p (68)
SLC13A1	solute carrier family 13, member 1	Enhanced	72,8	KUPKB	g,p,d,c					k, p (69)
ALDH8A1	aldehyde dehydrogenase 8 family, member A1	Group enriched	70,7			KUPKB	k	KUPKB		k, (70)
SLC36A2	solute carrier family 36, member 2	Highly enriched	63,9					KUPKB, UEPD	k/u	k, p (71)
SLC16A12	solute carrier family 16, member 12	Moderately enriched	61,7	KUPKB	p					k, p (72)
DAO	D-amino-acid oxidase	Group enriched	58,8			KUPKB, HKUPP	g		k	k, p (73)
RGN	regucalcin (senescence marker protein-30)	Group enriched	56,5	KUPKB	k (n.s)				k/u	k, p (74)
SLC7A9	solute carrier family 7, member 9	Group enriched	52,2							k, p (75)
SFXN2	sideroflexin 2	Enhanced	52						k	
SLC5A2	solute carrier family 5, member 2	Moderately enriched	49,5	KUPKB	k (n.s)			UEPD	k/u	k, p (76)
SLC5A10	solute carrier family 5, member 10	Moderately enriched	48					UEPD	k/u	k, (77)
ACY3	aspartoacylase (aminocyclase) 3	Enhanced	47,7					KUPKB, UEPD	k/u	k, (78)
SLC22A7	solute carrier family 22, member 7	Group enriched	47,4	KUPKB	p, d					k, p (79)
CHDH	choline dehydrogenase	Enhanced	47,2			KUPKB	k		k	k, p (80)
SLC22A11	solute carrier family 22, member 11	Group enriched	45,1					UEPD	k/u	k, p (81)
SLC2A9	solute carrier family 2, member 9	Enhanced	44,8							k, p (82)
CYP4F2	cytochrome P450 superfamily	Group enriched	44,6						k	k, p (83)
SLC23A1	solute carrier family 23, member 1	Enhanced	44,3					UEPD	k/u	k, p (84)
IL17RB	interleukin 17 receptor B	Enhanced	40,8	KUPKB	g,p,d,c					k, (85)
SLC13A2	solute carrier family 13, member 2	Group enriched	40,1	KUPKB	p			UEPD	u	k, p (86)
C7orf10	chromosome 7 open reading frame 10	Enhanced	38,8	KUPKB	p					
MAPT	microtubule-associated protein tau	Group enriched	38,4			KUPKB, HKUPP	g		k	
PAQR5	progestin and adipoQ receptor family V	Enhanced	38							
UGT3A1	UDP glycosyltransferase 3, polypeptide A1	Enhanced	36,7							k, (87)
PAX2	paired box 2	Moderately enriched	36							k, p (88)
ASPA	aspartoacylase	Enhanced	34,4	KUPKB	k (n.s)	KUPKB, HKUPP	g		k/u	k, (89)
SLC28A1	solute carrier family 28, member 1	Group enriched	34,3						u	k, g+p (90)
PKLR	pyruvate kinase, liver and RBC	Enhanced	30,5			KUPKB, HKUPP	g	UEPD	k/u/pl	k, p (91)
MYOM3	myomesin family, member 3	Group enriched	30,3						pl	
ASPDH	aspartate dehydrogenase domain containing	Group enriched	30,2						k/u/pl	k, (92)
KMO	kynurenine 3-monooxygenase	Enhanced	29	KUPKB	p				k	k, (93)
REN	renin	Group enriched	25,3							k, g+p (94)
ENPP6	ectonucleotide pyrophosphatase/PDE 6	Enhanced	24,5	KUPKB	p			UEPD	u/pl	k, (95)
SLC6A12	solute carrier family 6, member 12	Group enriched	24	KUPKB	p					k, c (96)
PALM3	paralemmin 3	Enhanced	23,7							k, p+d (97)
CDH6	cadherin 6, type 2, K-cadherin	Enhanced	19,5	KUPKB	p			KUPKB	u/pl	k, (98)
SLC34A3	solute carrier family 34, member 3	Group enriched	18,1	KUPKB	p,d,c					k, p (99)
ABCC2	ATP-binding cassette, sub-family C, member 2	Group enriched	16	KUPKB	k (n.s)					k, p (100)
SLC10A2	solute carrier family 10, member 2	Group enriched	12,3							k, p (101)
SLC22A13	solute carrier family 22, member 13	Highly enriched	11,7					UEPD		k, p (102)
LYG1	lysozyme G-like 1	Group enriched	10,6	KUPKB	p					
SLC6A18	solute carrier family 6, member 18	Highly enriched	10,6							k, p (103)
MNS1	meiosis-specific nuclear structural 1	Group enriched	9,8							k, (104)
RP11-407N17.3	Cutaneous T-cell lymphoma-related antigen 5	Enhanced	9,6							
RNF183	ring finger protein 183	Enhanced	8,3							

**Proximal tubuli elevated genes (continued)**										
ABCA4	ATP-binding cassette, sub-family A, member 4	Enhanced	7,4							
ARSF	arylsulfatase F	Enhanced	5,9					KUPKB, UEPD	u	
SLC5A11	solute carrier family 5, member 11	Group enriched	5,8							k, p (105)
FAM131C	family with sequence similarity 131, member C	Enhanced	3,3	KUPKB	p					
PNMA6A	paraneoplastic Ma antigen family member 6A	Enhanced	3,2	KUPKB	p					
C9orf135	chromosome 9 open reading frame 135	Group enriched	3,1							
RIPPLY1	ripply1 homolog (zebrafish)	Group enriched	2,5							
CALCR	calcitonin receptor	Enhanced	1,6							k, (106)
INSRR	insulin receptor-related receptor	Enhanced	1,5							k, p+d (107)
**c. distal tubuli elevated genes**										
UMOD	uromodulin	Highly enriched	1420,8	KUPKB	g,p,d,c	KUPKB, HKUPP	g	KUPKB, UEPD	k/u/pl	k, d (108)
SLC12A1	solute carrier family 12, member 1	Highly enriched	406,1	KUPKB	p,d,c			KUPKB, UEPD	k/u	k, d (109)
CALB1	calbindin 1, 28kDa	Moderately enriched	229,6	KUPKB	g,p,d,c	KUPKB, HKUPP	g	KUPKB, UEPD	k/u	k, d (110)
KCNJ1	potassium channel, subfamily J, member 1	Highly enriched	132	KUPKB	k (n.s)					k, d (111)
SLC12A3	solute carrier family 12, member 3	Highly enriched	129,3	KUPKB	p,d,c			KUPKB, UEPD	k/u	k, d (112)
CLDN8	claudin 8	Enhanced	56,9	KUPKB	p,d,c					k, d (113)
CASR	calcium-sensing receptor	Enhanced	21,1	KUPKB	k (n.s)					k, d (114)
GLTPD2	glycolipid transfer protein domain containing 2	Enhanced	12,5						pl	k, (115)
TFAP2B	transcription factor AP-2 beta	Group enriched	8,5						k	k, d+c (116)
**d. Collecting duct elevated genes (* only intercaleted cells)**										
AQP2	aquaporin 2 (collecting duct)	Highly enriched	314,7	KUPKB	g,p,d,c			KUPKB, UEPD	u	k, c (117)
HPN *	hepsin	Enhanced	91,6	KUPKB	p					k, (118)
TMEM213*	transmembrane protein 213	Group enriched	65,3							
ATP6V0D2 *	ATPase, H+ transporting, V0 subunit d2	Moderately enriched	40,6			KUPKB, HKUPP	g			k, (119)
PVALB *	parvalbumin	Moderately enriched	40	KUPKB	k (n.s)			KUPKB		k, d (120)
RHBG	Rh family, B glycoprotein (gene/pseudogene)	Group enriched	15,3	KUPKB	p,d,c					k, d+c (121)
SLC4A9	solute carrier family 4, member 9	Moderately enriched	11,3	KUPKB	p,d,c					k, c (122)
CLNK *	cytokine-dependent hematopoietic cell linker	Enhanced	3,6							

* indicates proteins that are only found in the subsegment of intercalated cells of collecting duct.

Farrah (1) – Report of compiled MS datasets for blood, urine and kidney tissue. Farah T. *et al* (J Proteome Res. 2014 Jan 3;13(1):60-75)

'literature' – Previous publication that has characterised the protein in kidney cells or tissue.

Abbreviations:
K; kidney, g; glomeruli, p; proximal tubuli, d; distal tubuli, c; collecting duct; pl:plasma; u: urine
KUPKB; Kidney and Urinary Pathway Knowledge base (http://www.kupkb.org/). Omics datasets that have been extracted from scientific publications and other related renal databases.
HKUPP; Human Kidney and Urinary Proteome Project (http://www.hkupp.org/). Datasets of identified proteins localized in isolated human glomeruli using 2D SDS-PAGE and LC-MS/MS.
UEPD; Urinary Exosome Protein database (http://dir.nhlbi.nih.gov/papers/lkem/exosome/). Datasets of human urinary exosome proteins based on published and unpublished protein mass spectrometry data from the NHLBI Laboratory of Kidney and Electrolyte Metabolism.

Supplementary Table S4 - References

No	Title	URL	Description	Details	ShortDetails	Resource	Type	Identifiers	Db	EntrezUID	Properties
1	State of the human proteome in 2013 as viewed through PeptideAtlas: comparing the kidney, urine, and plasma proteomes for the biology- and disease-driven Human Proteome Project.	/pubmed/24261998	Farrah T, Deutsch EW, Omenn GS, Sun Z, Watts JD, Yamamoto T, Shteynberg D, Harris MM, Moritz RL.	J Proteome Res. 2014 Jan 3;13(1):60-75. doi: 10.1021/pr4010037. Epub 2013 Dec 6.	J Proteome Res. 2014	PubMed	citation	PMID:24261998 | PMCID:PMC3951210	pubmed	24261998	create date:2013/11/23 | first author:Farrah T
2	Sulfate contributes to the negative charge of podocalyxin, the major sialoglycoprotein of the glomerular filtration slits.	/pubmed/2052617	Dekan G, Gabel C, Farquhar MG.	Proc Natl Acad Sci U S A. 1991 Jun 15;88(12):5398-402.	Proc Natl Acad Sci U S A. 1991	PubMed	citation	PMID:2052617 | PMCID:PMC51880	pubmed	2052617	create date:1991/06/15 | first author:Dekan G
3	FGF-1 in normal and regenerating kidney: expression in mononuclear, interstitial, and regenerating epithelial cells.	/pubmed/7503231	Ichimura T, Maier JA, Maciag T, Zhang G, Stevens JL.	Am J Physiol. 1995 Nov;269(5 Pt 2):F653-62.	Am J Physiol. 1995	PubMed	citation	PMID:7503231	pubmed	7503231	create date:1995/11/01 | first author:Ichimura T
4	Podocin localizes in the kidney to the slit diaphragm area.	/pubmed/11786407	Roselli S, Gribouval O, Boute N, Sich M, Benessy F, Atti テゥ T, Gubler MC, Antignac C.	Am J Pathol. 2002 Jan;160(1):131-9.	Am J Pathol. 2002	PubMed	citation	PMID:11786407 | PMCID:PMC1867125	pubmed	11786407	create date:2002/01/12 | first author:Roselli S
5	Novel expression of sodium/myo-inositol co-transporter in podocytes in puromycin aminonucleoside nephrosis.	/pubmed/15031335	Watanabe Y, Kobayashi T, Yaoita E, Kawachi H, Yamauchi A, Inoue T, Shimizu F, Yoshida Y, El-Shemi AG, Okada H, Suzuki H, Yamamoto T.	Nephrol Dial Transplant. 2004 Apr;19(4):817-22.	Nephrol Dial Transplant. 2004	PubMed	citation	PMID:15031335	pubmed	15031335	create date:2004/03/20 | first author:Watanabe Y
6	Distribution of parathyroid hormone-2 receptor messenger ribonucleic acid in rat.	/pubmed/8828488	Usdin TB, Bonner TI, Harta G, Mezey E.	Endocrinology. 1996 Oct;137(10):4285-97.	Endocrinology. 1996	PubMed	citation	PMID:8828488	pubmed	8828488	create date:1996/10/01 | first author:Usdin TB
7	Orphan receptor GPR110, an oncogene overexpressed in lung and prostate cancer.	/pubmed/20149256	Lum AM, Wang BB, Beck-Engeser GB, Li L, Channa N, Wabl M.	BMC Cancer. 2010 Feb 11;10:40. doi: 10.1186/1471-2407-10-40.	BMC Cancer. 2010	PubMed	citation	PMID:20149256 | PMCID:PMC2830182	pubmed	20149256	create date:2010/02/13 | first author:Lum AM
8	Nephrin is specifically located at the slit diaphragm of glomerular podocytes.	/pubmed/10393930	Ruotsalainen V, Ljungberg P, Wartiovaara J, Lenkkeri U, Kestil テ、 M, Jalanko H, Holmberg C, Tryggvason K.	Proc Natl Acad Sci U S A. 1999 Jul 6;96(14):7962-7.	Proc Natl Acad Sci U S A. 1999	PubMed	citation	PMID:10393930 | PMCID:PMC22170	pubmed	10393930	create date:1999/07/08 | first author:Ruotsalainen V
9	Molecular cloning of cDNAs encoding human GLEPP1, a membrane protein tyrosine phosphatase: characterization of the GLEPP1 protein distribution in human kidney and assignment of the GLEPP1 gene to human chromosome 12p12-p13.	/pubmed/7665166	Wiggins RC, Wiggins JE, Goyal M, Wharram BL, Thomas PE.	Genomics. 1995 May 1;27(1):174-81.	Genomics. 1995	PubMed	citation	PMID:7665166	pubmed	7665166	create date:1995/05/01 | first author:Wiggins RC
10	Characterization of the Crumbs homolog 2 (CRB2) gene and analysis of its role in retinitis pigmentosa and Leber congenital amaurosis.	/pubmed/15851977	van den Hurk JA, Rashbass P, Roepman R, Davis J, Voesenek KE, Arends ML, Zonneveld MN, van Roekel MH, Cameron K, Rohrschneider K, Heckenlively JR, Koenekoop RK, Hoyng CB, Cremers FP, den Hollander AI.	Mol Vis. 2005 Apr 15;11:263-73.	Mol Vis. 2005	PubMed	citation	PMID:15851977	pubmed	15851977	create date:2005/04/27 | first author:van den Hurk JA
11	NKCC2 surface expression in mammalian cells: down-regulation by novel interaction with aldolase B.	/pubmed/17848580	Benziane B, Demaretz S, Defontaine N, Zaarour N, Cheval L, Bourgeois S, Klein C, Froissart M, Blanchard A, Paillard M, Gamba G, Houillier P, Laghmani K.	J Biol Chem. 2007 Nov 16;282(46):33817-30. Epub 2007 Sep 11.	J Biol Chem. 2007	PubMed	citation	PMID:17848580	pubmed	17848580	create date:2007/09/13 | first author:Benziane B
12	Integrated transcriptomic and proteomic evaluation of gentamicin nephrotoxicity in rats.	/pubmed/22061828	Com E, Boitier E, Marchandeau JP, Brandenburg A, Schroeder S, Hoffmann D, Mally A, Gautier JC.	Toxicol Appl Pharmacol. 2012 Jan 1;258(1):124-33. doi: 10.1016/j.taap.2011.10.015. Epub 2011 Oct 29.	Toxicol Appl Pharmacol. 2012	PubMed	citation	PMID:22061828	pubmed	22061828	create date:2011/11/09 | first author:Com E
13	Interactions of MAP17 with the NaPi-IIa/PDZK1 protein complex in renal proximal tubular cells.	/pubmed/12837682	Pribanic S, Gisler SM, Bacic D, Madjdpour C, Hernando N, Sorribas V, Gantenbein A, Biber J, Murer H.	Am J Physiol Renal Physiol. 2003 Oct;285(4):F784-91. Epub 2003 Jul 1.	Am J Physiol Renal Physiol. 2003	PubMed	citation	PMID:12837682	pubmed	12837682	create date:2003/07/03 | first author:Pribanic S

14 Expression and function of arginine-producing and consuming-enzymes in the kidney. /pubmed/21567240 Levillain O. Amino Acids. 2012 Apr;42(4):1237-52. doi: 10.1007/s00726-011-0897-z. Epub 2011 May 13. Amino Acids. 2012 PubMed citation PMID:21567240 pubmed 21567240 create date:2011/05/14 | first author:Levillain O

15 Development of an immunoassay for the kidney-specific protein myo-inositol oxygenase, a potential biomarker of acute kidney injury./pubmed/24486646 Gaut JP, Crimmins DL, Ohlendorf MF, Lockwood CM, Griest TA, Brada NA, Hoshi M, Sato B, Hotchkiss RS, Jain S, Ladenson JH. Clin Chem. 2014 May;60(5):747-57. doi: 10.1373/clinchem.2013.212993. Epub 2014 Jan 31. Clin Chem. 2014 PubMed citation PMID:24486646 | PMCID:PMC4128578 pubmed 24486646 create date:2014/02/04 | first author:Gaut JP

16 The role of reversible phosphorylation in the hormonal control of phenylalanine hydroxylase in isolated rat proximal kidney tubules. /pubmed/8389135 Richardson SC, Aspbury RA, Fisher MJ. Biochem J. 1993 Jun 1;292 (Pt 2):419-24. Biochem J. 1993 PubMed citation PMID:8389135 | PMCID:PMC1134225 pubmed 8389135 create date:1993/06/01 | first author:Richardson SC

17 Immunohistochemical detection of betaine-homocysteine S-methyltransferase in human, pig, and rat liver and kidney. /pubmed/11516176 Delgado-Reyes CV, Wallig MA, Garrow TA. Arch Biochem Biophys. 2001 Sep 1;393(1):184-6.Arch Biochem Biophys. 2001 PubMed citation PMID:11516176 pubmed 11516176 create date:2001/08/23 | first author:Delgado-Reyes CV

18 N-acetyltransferase 8, a positional candidate for blood pressure and renal regulation: resequencing, association and in silico study. /pubmed/18402670 Juhanson P, Kepp K, Org E, Veldre G, Kelgo P, Rosenberg M, Viigimaa M, Laan M. BMC Med Genet. 2008 Apr 10;9:25. doi: 10.1186/1471-2350-9-25. BMC Med Genet. 2008 PubMed citation PMID:18402670 | PMCID:PMC2330028 pubmed 18402670 create date:2008/04/12 | first author:Juhanson P

19 Role of angiotensin II in the enhancement of ammonia production and secretion by the proximal tubule in metabolic acidosis. /pubmed/18287403 Nagami GT. Am J Physiol Renal Physiol. 2008 Apr;294(4):F874-80. doi: 10.1152/ajprenal.00286.2007. Epub 2008 Feb 20. Am J Physiol Renal Physiol. 2008 PubMed citation PMID:18287403 pubmed 18287403 create date:2008/02/22 | first author:Nagami GT

20 High expression of bovine alpha glutathione S-transferase (GSTA1, GSTA2) subunits is mainly associated with steroidogenically active cells and regulated by gonadotropins in bovine ovarian follicles. /pubmed/10433206 Rabahi F, Br テサ l テゥ S, Sirois J, Beckers JF, Silversides DW, Lussier JG. Endocrinology. 1999 Aug;140(8):3507-17. Endocrinology. 1999 PubMed citation PMID:10433206 pubmed 10433206 create date:1999/08/05 | first author:Rabahi F

21 PDZK1: I. a major scaffolder in brush borders of proximal tubular cells. /pubmed/14531806 Gisler SM, Pribanic S, Bacic D, Forrer P, Gantenbein A, Sabourin LA, Tsuji A, Zhao ZS, Manser E, Biber J, Murer H. Kidney Int. 2003 Nov;64(5):1733-45. Kidney Int. 2003 PubMed citation PMID:14531806 pubmed 14531806 create date:2003/10/09 | first author:Gisler SM

22 N-acetylamino acid utilization by kidney aminoacylase-1. /pubmed/18222180 Lindner HA, T テ、fler-Naumann M, R テカ hm KH. Biochimie. 2008 May;90(5):773-80. doi: 10.1016/j.biochi.2007.12.006. Epub 2008 Jan 5. Biochimie. 2008 PubMed citation PMID:18222180 pubmed 18222180 create date:2008/01/29 | first author:Lindner HA

23 Betaine-homocysteine methyltransferase-2: cDNA cloning, gene sequence, physical mapping, and expression of the human and mouse genes. /pubmed/11087663 Chadwick LH, McCandless SE, Silverman GL, Schwartz S, Westaway D, Nadeau JH. Genomics. 2000 Nov 15;70(1):66-73. Genomics. 2000 PubMed citation PMID:11087663 pubmed 11087663 create date:2000/11/23 | first author:Chadwick LH

24 The molecular basis of cystinuria. /pubmed/15499206 Goodyer P. Nephron Exp Nephrol. 2004;98(2):e45-9. Review. Nephron Exp Nephrol. 2004 PubMed citation PMID:15499206 pubmed 15499206 create date:2004/10/23 | first author:Goodyer P

25 The effect of a low-protein diet and dietary supplementation of threonine on tyrosine and 2-(2-nitro-4-trifluoromethylbenzoyl) cyclohexane-1,3-dione-induced corneal lesions, the extent of tyrosinemia, and the activity of enzymes involved in tyrosine catabolism in the rat. /pubmed/9630461 Lock EA, Gaskin P, Ellis MK, Robinson M, Provan WM, Smith LL. Toxicol Appl Pharmacol. 1998 May;150(1):125-32. Toxicol Appl Pharmacol. 1998 PubMed citation PMID:9630461 pubmed 9630461 create date:1998/06/19 | first author:Lock EA

26 Sodium-coupled dicarboxylate and citrate transporters from the SLC13 family. /pubmed/24114175 Pajor AM. Pflugers Arch. 2014 Jan;466(1):119-30. doi: 10.1007/s00424-013-1369-y. Epub 2013 Oct 10. Review. Pflugers Arch. 2014 PubMed citation PMID:24114175 pubmed 24114175 create date:2013/10/12 | first author:Pajor AM

27 Purification, characterization and differentiation-dependent expression of a perchloric acid soluble protein from rat kidney. /pubmed/9609467 Asagi K, Oka T, Arao K, Suzuki I, Thakur MK, Izumi K, Natori Y. Nephron. 1998;79(1):80-90. Nephron. 1998 PubMed citation PMID:9609467 pubmed 9609467 create date:1998/06/03 | first author:Asagi K

28 Expression and cellular localization of kininogens in the human kidney. /pubmed/8807575 Hermann A, Braun A, Figueroa CD, M テシ ller-Esterl W, Fritz H, Rehbock J. Kidney Int. 1996 Jul;50(1):79-84. Kidney Int. 1996 PubMed citation PMID:8807575 pubmed 8807575 create date:1996/07/01 | first author:Hermann A

29 Sex-dependent expression of Oat3 (Slc22a8) and Oat1 (Slc22a6) proteins in murine kidneys. /pubmed/23389457 Breljak D, Brzica H, Sweet DH, Anzai N, Sabolic I. Am J Physiol Renal Physiol. 2013 Apr 15;304(8):F1114-26. doi: 10.1152/ajprenal.00201.2012. Epub 2013 Feb 6. Am J Physiol Renal Physiol. 2013 PubMed citation PMID:23389457 | PMCID:PMC3625837 pubmed 23389457 create date:2013/02/08 | first author:Breljak D

30 Designation of enzyme activity of glycine-N-acyltransferase family genes and depression of glycine-N-acyltransferase in human hepatocellular carcinoma. /pubmed/22475485 Matsuo M, Terai K, Kameda N, Matsumoto A, Kurokawa Y, Funase Y, Nishikawa K, Sugaya N, Hiruta N, Kishimoto T. Biochem Biophys Res Commun. 2012 Apr 20;420(4):901-6. doi: 10.1016/j.bbrc.2012.03.099. Epub 2012 Mar 27. Biochem Biophys Res Commun. 2012 PubMed citation PMID:22475485 pubmed 22475485 create date:2012/04/06 | first author:Matsuo M

31 Proteomic analysis of clear cell renal cell carcinoma. Identification of potential tumor markers. /pubmed/20464042 Sun CY, Zang YC, San YX, Sun W, Zhang L. Saudi Med J. 2010 May;31(5):525-32. Saudi Med J. 2010 PubMed citation PMID:20464042 pubmed 20464042 create date:2010/05/14 | first author:Sun CY

32 Genomic structure, alternative maturation and tissue expression of the human BBOX1 gene. /pubmed/17110165 Rigault C, Le Borgne F, Demarquoy J. Biochim Biophys Acta. 2006 Dec;1761(12):1469-81. Epub 2006 Oct 4. Biochim Biophys Acta. 2006 PubMed citation PMID:17110165 pubmed 17110165 create date:2006/11/18 | first author:Rigault C

33 Four distinct membrane-bound dipeptidase RNAs are differentially expressed and show discordant regulation with gamma-glutamyl transpeptidase. /pubmed/8663190 Habib GM, Barrios R, Shi ZZ, Lieberman MW. J Biol Chem. 1996 Jul 5;271(27):16273-80. J Biol Chem. 1996 PubMed citation PMID:8663190 pubmed 8663190 create date:1996/07/05 | first author:Habib GM

34 Different hydrolases involved in bioactivation of prodrug-type angiotensin receptor blockers: carboxymethylenebutenolidase and carboxylesterase 1. /pubmed/23946449 Ishizuka T, Yoshigae Y, Murayama N, Izumi T. Drug Metab Dispos. 2013 Nov;41(11):1888-95. doi: 10.1124/dmd.113.053595. Epub 2013 Aug 14. Drug Metab Dispos. 2013 PubMed citation PMID:23946449 pubmed 23946449 create date:2013/08/16 | first author:Ishizuka T

35 Designation of enzyme activity of glycine-N-acyltransferase family genes and depression of glycine-N-acyltransferase in human hepatocellular carcinoma. /pubmed/22475485 Matsuo M, Terai K, Kameda N, Matsumoto A, Kurokawa Y, Funase Y, Nishikawa K, Sugaya N, Hiruta N, Kishimoto T. Biochem Biophys Res Commun. 2012 Apr 20;420(4):901-6. doi: 10.1016/j.bbrc.2012.03.099. Epub 2012 Mar 27. Biochem Biophys Res Commun. 2012 PubMed citation PMID:22475485 pubmed 22475485 create date:2012/04/06 | first author:Matsuo M

36 Renal dopaminergic mechanisms in renal parenchymal diseases and hypertension. /pubmed/11369822 Pestana M, Jardim H, Correia F, Vieira-Coelho MA, Soares-da-Silva P. Nephrol Dial Transplant. 2001;16 Suppl 1:53-9. Review. Nephrol Dial Transplant. 2001 PubMed citation PMID:11369822 pubmed 11369822 create date:2001/05/23 | first author:Pestana M

37 Metabolic effects of large fructose loads in different parts of the rat nephron. /pubmed/6773936 Burch HB, Choi S, Dence CN, Alvey TR, Cole BR, Lowry OH. J Biol Chem. 1980 Sep 10;255(17):8239-44. J Biol Chem. 1980 PubMed citation PMID:6773936 pubmed 6773936 create date:1980/09/10 | first author:Burch HB

38 SLC7A7, encoding a putative permease-related protein, is mutated in patients with lysinuric protein intolerance. /pubmed/10080183 Borsani G, Bassi MT, Sperandeo MP, De Grandi A, Buoninconti A, Riboni M, Manzoni M, Incerti B, Pepe A, Andria G, Ballabio A, Sebastio G. Nat Genet. 1999 Mar;21(3):297-301. Nat Genet. 1999 PubMed citation PMID:10080183 pubmed 10080183 create date:1999/03/18 | first author:Borsani G

39 Differential localization of flavin-containing monooxygenase (FMO) isoforms 1, 3, and 4 in rat liver and kidney and evidence for expression of FMO4 in mouse, rat, and human liver and kidney microsomes. /pubmed/19307449 Novick RM, Mitzey AM, Brownfield MS, Elfarra AA. J Pharmacol Exp Ther. 2009 Jun;329(3):1148-55. doi: 10.1124/jpet.109.152058. Epub 2009 Mar 23. J Pharmacol Exp Ther. 2009 PubMed citation PMID:19307449 | PMCID:PMC2683785 pubmed 19307449 create date:2009/03/25 | first author:Novick RM

40 Serine isotopmer analysis by 13C-NMR defines glycine-serine interconversion in situ in the renal proximal tubule. /pubmed/9244172 Cowin GJ, Willgoss DA, Bartley J, Endre ZH. Biochim Biophys Acta. 1996 Jan 10;1310(1):32-40. Biochim Biophys Acta. 1996 PubMed citation PMID:9244172 pubmed 9244172 create date:1997/01/10 | first author:Cowin GJ

41 A novel gene family defined by human dihydropyrimidinase and three related proteins with differential tissue distribution. /pubmed/8973361 Hamajima N, Matsuda K, Sakata S, Tamaki N, Sasaki M, Nonaka M. Gene. 1996 Nov 21;180(1-2):157-63. Gene. 1996 PubMed citation PMID:8973361 pubmed 8973361 create date:1996/11/21 | first author:Hamajima N

42 The SLC34 family of sodium-dependent phosphate transporters. /pubmed/24352629 Wagner CA, Hernando N, Forster IC, Biber J. Pflugers Arch. 2014 Jan;466(1):139-53. doi: 10.1007/s00424-013-1418-6. Epub 2013 Dec 19. Review. Pflugers Arch. 2014 PubMed citation PMID:24352629 pubmed 24352629 create date:2013/12/20 | first author:Wagner CA

43 Human mitochondrial phosphoenolpyruvate carboxykinase 2 gene. Structure, chromosomal localization and tissue-specific expression. /pubmed/9657976 Modaressi S, Brechtel K, Christ B, Jungermann K. Biochem J. 1998 Jul 15;333 (Pt 2):359-66. Biochem J. 1998 PubMed citation PMID:9657976 | PMCID:PMC1219593 pubmed 9657976 create date:1998/07/11 | first author:Modaressi S

44 SLC7A8, a gene mapping within the lysinuric protein intolerance critical region, encodes a new member of the glycoprotein-associated amino acid transporter family. /pubmed/10610726 Bassi MT, Sperandeo MP, Incerti B, Bulfone A, Pepe A, Surace EM, Gattuso C, De Grandi A, Buoninconti A, Riboni M, Manzoni M, Andria G, Ballabio A, Borsani G, Sebastio G. Genomics. 1999 Dec 1;62(2):297-303. Genomics. 1999 PubMed citation PMID:10610726 pubmed 10610726 create date:1999/12/28 | first author:Bassi MT

45 Cloning and characterization of human FTCD on 21q22.3, a candidate gene for glutamate formiminotransferase deficiency./pubmed/10773664 Solans A, Estivill X, de la Luna S. Cytogenet Cell Genet. 2000;88(1-2):43-9. Cytogenet Cell Genet. 2000 PubMed citation PMID:10773664 pubmed 10773664 create date:2000/04/25 | first author:Solans A

46 Human agmatinase is diminished in the clear cell type of renal cell carcinoma. /pubmed/14648699 Dallmann K, Junker H, Balabanov S, Zimmermann U, Giebel J, Walther R. Int J Cancer. 2004 Jan 20;108(3):342-7. Int J Cancer. 2004 PubMed citation PMID:14648699 pubmed 14648699 create date:2003/12/04 | first author:Dallmann K

47 Tissue specificity of rat mitochondrial dimethylglycine dehydrogenase expression. /pubmed/7513513 Lang H, Minaian K, Freudenberg N, Hoffmann R, Brandsch R. Biochem J. 1994 Apr 15;299 (Pt 2):393-8. Biochem J. 1994 PubMed citation PMID:7513513 | PMCID:PMC1138285 pubmed 7513513 create date:1994/04/15 | first author:Lang H

48 Localization of adenylate kinase 4 in mouse tissues. /pubmed/19492028 Miyoshi K, Akazawa Y, Horiguchi T, Noma T. Acta Histochem Cytochem. 2009 Apr 28;42(2):55-64. doi: 10.1267/ahc.08012. Epub 2009 Apr 7. Acta Histochem Cytochem. 2009 PubMed citation PMID:19492028 | PMCID:PMC2685024 pubmed 19492028 create date:2009/06/06 | first author:Miyoshi K

49 gamma-Glutamyl transpeptidase catalyses the extracellular detoxification of cisplatin in a human cell line derived from the proximal convoluted tubule of the kidney. /pubmed/12706370 Paolicchi A, Sotiropuolou M, Perego P, Daubeuf S, Visvikis A, Lorenzini E, Franzini M, Romiti N, Chieli E, Leone R, Apostoli P, Colangelo D, Zunino F, Pompella A. Eur J Cancer. 2003 May;39(7):996-1003. Eur J Cancer. 2003 PubMed citation PMID:12706370 pubmed 12706370 create date:2003/04/23 | first author:Paolicchi A

50 Klotho: a novel phosphaturic substance acting as an autocrine enzyme in the renal proximal tubule. /pubmed/20466874 Hu MC, Shi M, Zhang J, Pastor J, Nakatani T, Lanske B, Razzaque MS, Rosenblatt KP, Baum MG, Kuro-o M, Moe OW. FASEB J. 2010 Sep;24(9):3438-50. doi: 10.1096/fj.10-154765. Epub 2010 May 13. FASEB J. 2010 PubMed citation PMID:20466874 | PMCID:PMC2923354 pubmed 20466874 create date:2010/05/15 | first author:Hu MC

51 Mouse amnionless, which is required for primitive streak assembly, mediates cell-surface localization and endocytic function of cubilin on visceral endoderm and kidney proximal tubules. /pubmed/15342463 Strope S, Rivi R, Metzger T, Manova K, Lacy E. Development. 2004 Oct;131(19):4787-95. Epub 2004 Sep 1. Erratum in: Development. 2004 Oct;131(4):5211. Development. 2004 PubMed citation PMID:15342463 pubmed 15342463 create date:2004/09/03 | first author:Strope S

52 Mistargeting of peroxisomal EHHADH and inherited renal Fanconi's syndrome. /pubmed/24401050 Klootwijk ED, Reichold M, Helip-Wooley A, Tolaymat A, Broeker C, Robinette SL, Reinders J, Peindl D, Renner K, Eberhart K, Assmann N, Oefner PJ, Dettmer K, Sterner C, Schroeder J, Zorger N, Witzgall R, Reinhold SW, Stanescu HC, Bockenhauer D, Jaureguiberry G, Courtneidge H, et al. N Engl J Med. 2014 Jan 9;370(2):129-38. doi: 10.1056/NEJMoa1307581. N Engl J Med. 2014 PubMed citation PMID:24401050 pubmed 24401050 create date:2014/01/10 | first author:Klootwijk ED

53 Human alanine-glyoxylate aminotransferase 2 lowers asymmetric dimethylarginine and protects from inhibition of nitric oxide production. /pubmed/20018850 Rodionov RN, Murry DJ, Vaulman SF, Stevens JW, Lentz SR. J Biol Chem. 2010 Feb 19;285(8):5385-91. doi: 10.1074/jbc.M109.091280. Epub 2009 Dec 14. J Biol Chem. 2010 PubMed citation PMID:20018850 | PMCID:PMC2820767 pubmed 20018850 create date:2009/12/19 | first author:Rodionov RN

54 A novel superoxide-producing NAD(P)H oxidase in kidney. /pubmed/11032835 Shiose A, Kuroda J, Tsuruya K, Hirai M, Hirakata H, Naito S, Hattori M, Sakaki Y, Sumimoto H. J Biol Chem. 2001 Jan 12;276(2):1417-23. J Biol Chem. 2001 PubMed citation PMID:11032835 pubmed 11032835 create date:2000/10/18 | first author:Shiose A

55 Apolipoprotein M: progress in understanding its regulation and metabolic functions. /pubmed/17101555 Christoffersen C, Dahlb ﾃ､ck B, Nielsen LB. Scand J Clin Lab Invest. 2006;66(7):631-7. Review. Scand J Clin Lab Invest. 2006 PubMed citation PMID:17101555 pubmed 17101555 create date:2006/11/15 | first author:Christoffersen C

56 Characterization of monocarboxylate transport in human kidney HK-2 cells. /pubmed/17140255 Wang Q, Lu Y, Yuan M, Darling IM, Repasky EA, Morris ME. Mol Pharm. 2006 Nov-Dec;3(6):675-85. Mol Pharm. 2006 PubMed citation PMID:17140255 pubmed 17140255 create date:2006/12/05 | first author:Wang Q

57 Renal transport of uric acid: evolving concepts and uncertainties. /pubmed/23089270 Bobulescu IA, Moe OW. Adv Chronic Kidney Dis. 2012 Nov;19(6):358-71. doi: 10.1053/j.ackd.2012.07.009. Review. Adv Chronic Kidney Dis. 2012 PubMed citation PMID:23089270 | PMCID:PMC3619397 pubmed 23089270 create date:2012/10/24 | first author:Bobulescu IA

58 Changes in kynurenine pathway metabolism in the brain, liver and kidney of aged female Wistar rats. /pubmed/22032336 Braidy N, Guillemin GJ, Mansour H, Chan-Ling T, Grant R. FEBS J. 2011 Nov;278(22):4425-34. doi: 10.1111/j.1742-4658.2011.08366.x. FEBS J. 2011 PubMed citation PMID:22032336 pubmed 22032336 create date:2011/10/29 | first author:Braidy N

59 Common missense variant of monocarboxylate transporter 9 (MCT9/SLC16A9) gene is associated with renal overload gout, but not with all gout susceptibility. /pubmed/23990105 Nakayama A, Matsuo H, Shimizu T, Ogata H, Takada Y, Nakashima H, Nakamura T, Shimizu S, Chiba T, Sakiyama M, Ushiyama C, Takada T, Inoue K, Kawai S, Hishida A, Wakai K, Hamajima N, Ichida K, Sakurai Y, Kato Y, Shimizu T, Shinomiya N. Hum Cell. 2013 Dec;26(4):133-6. doi: 10.1007/s13577-013-0073-8. Epub 2013 Aug 29. Hum Cell. 2013 PubMed citation PMID:23990105 | PMCID:PMC3844819 pubmed 23990105 create date:2013/08/31 | first author:Nakayama A

60

Localization of NBC-1 variants in human kidney and renal cell carcinoma. /pubmed/14559244 Yamada H, Yamazaki S, Moriyama N, Hara C, Horita S, Enomoto Y, Kudo A, Kawakami H, Tanaka Y, Fujita T, Seki G. Biochem Biophys Res Commun. 2003 Oct 31;310(4):1213-8. Biochem Biophys Res Commun. 2003 PubMed citation PMID:14559244 pubmed 14559244 create date:2003/10/16 | first author:Yamada H

61 Structural organization of the human sorbitol dehydrogenase gene (SORD). /pubmed/7782086 Iwata T, Popescu NC, Zimonjic DB, Karlsson C, H ﾃｶﾃｶ g JO, Vaca G, Rodriguez IR, Carper D. Genomics. 1995 Mar 1;26(1):55-62. Genomics. 1995 PubMed citation PMID:7782086 pubmed 7782086 create date:1995/03/01 | first author:Iwata T

62 Human sodium phosphate transporter 4 (hNPT4/SLC17A3) as a common renal secretory pathway for drugs and urate. /pubmed/20810651 Jutabha P, Anzai N, Kitamura K, Taniguchi A, Kaneko S, Yan K, Yamada H, Shimada H, Kimura T, Katada T, Fukutomi T, Tomita K, Urano W, Yamanaka H, Seki G, Fujita T, Moriyama Y, Yamada A, Uchida S, Wempe MF, Endou H, Sakurai H. J Biol Chem. 2010 Nov

5;285(45):35123-32. doi: 10.1074/jbc.M110.121301. Epub 2010 Sep 1. J Biol Chem. 2010 PubMed citation PMID:20810651 | PMCID:PMC2966126 pubmed 20810651 create date:2010/09/03 | first author:Jutabha P

63 Human TMEM174 that is highly expressed in kidney tissue activates AP-1 and promotes cell proliferation. /pubmed/20331980 Wang P, Sun B, Hao D, Zhang X, Shi T, Ma D. Biochem Biophys Res Commun. 2010 Apr 16;394(4):993-9. doi: 10.1016/j.bbrc.2010.03.109. Epub 2010 Mar 21. Erratum in: Biochem Biophys Res Commun. 2011 Jan 7;404(1):580. Biochem Biophys Res Commun. 2010 PubMed citation PMID:20331980 pubmed 20331980 create date:2010/03/25 | first author:Wang P

64 Novel renal amino acid transporters. /pubmed/15709970 Verrey F, Ristic Z, Romeo E, Ramadan T, Makrides V, Dave MH, Wagner CA, Camargo SM. Annu Rev Physiol. 2005;67:557-72. Review. Annu Rev Physiol. 2005 PubMed citation PMID:15709970 pubmed 15709970 create date:2005/02/16 | first author:Verrey F

65 Triglyceride accumulation in injured renal tubular cells: alterations in both synthetic and catabolic pathways. /pubmed/15882263 Johnson AC, Stahl A, Zager RA. Kidney Int. 2005 Jun;67(6):2196-209. Kidney Int. 2005 PubMed citation PMID:15882263 pubmed 15882263 create date:2005/05/11 | first author:Johnson AC

66 Identification and expression of a cDNA encoding human alpha-amino-beta-carboxymuconate-epsilon-semialdehyde decarboxylase (ACMSD). A key enzyme for the tryptophan-niacine pathway and quinolinate hypothesis"." /pubmed/12140278 Fukuoka S, Ishiguro K, Yanagihara K, Tanabe A, Egashira Y, Sanada H, Shibata K. J Biol Chem. 2002 Sep 20;277(38):35162-7. Epub 2002 Jul 24. J Biol Chem. 2002 PubMed citation PMID:12140278 pubmed 12140278 create date:2002/07/26 | first author:Fukuoka S

67 Transcriptional analysis of the molecular basis of human kidney aging using cDNA microarray profiling. /pubmed/16316342 Melk A, Mansfield ES, Hsieh SC, Hernandez-Boussard T, Grimm P, Rayner DC, Halloran PF, Sarwal MM. Kidney Int. 2005 Dec;68(6):2667-79. Kidney Int. 2005 PubMed citation PMID:16316342 pubmed 16316342 create date:2005/12/01 | first author:Melk A

68 Gene expression analysis defines the proximal tubule as the compartment for endocytic receptor-mediated uptake in the Xenopus pronephric kidney. /pubmed/18551302 Christensen EI, Raciti D, Reggiani L, Verroust PJ, Br テ､ndli AW. Pflugers Arch. 2008 Sep;456(6):1163-76. doi: 10.1007/s00424-008-0488-3. Epub 2008 Jun 13. Pflugers Arch. 2008 PubMed citation PMID:18551302 pubmed 18551302 create date:2008/06/14 | first author:Christensen EI

69 Na+-sulfate cotransporter SLC13A1. /pubmed/24193406 Markovich D. Pflugers Arch. 2014 Jan;466(1):131-7. doi: 10.1007/s00424-013-1388-8. Epub 2013 Nov 6. Review. Pflugers Arch. 2014 PubMed citation PMID:24193406 pubmed 24193406 create date:2013/11/07 | first author:Markovich D

70 Expression of retinoic acid-synthesizing and -metabolizing enzymes during nephrogenesis in the rat. /pubmed/15567713 Marlier A, Gilbert T. Gene Expr Patterns. 2004 Dec;5(2):179-85. Gene Expr Patterns. 2004 PubMed citation PMID:15567713 pubmed 15567713 create date:2004/11/30 | first author:Marlier A

71 Renal imino acid and glycine transport system ontogeny and involvement in developmental iminoglycinuria. /pubmed/20377526 Vanslambrouck JM, Br テカ er A, Thavyogarajah T, Holst J, Bailey CG, Br テカ er S, Rasko JE. Biochem J. 2010 May 27;428(3):397-407. doi: 10.1042/BJ20091667. Biochem J. 2010 PubMed citation PMID:20377526 pubmed 20377526 create date:2010/04/10 | first author:Vanslambrouck JM

72 Mutation of solute carrier SLC16A12 associates with a syndrome combining juvenile cataract with microcornea and renal glucosuria. /pubmed/18304496 Kloeckener-Gruissem B, Vandekerckhove K, N テシ rnberg G, Neidhardt J, Zeitz C, N テシ rnberg P, Schipper I, Berger W. Am J Hum Genet. 2008 Mar;82(3):772-9. doi: 10.1016/j.ajhg.2007.12.013. Epub 2008 Feb 14. Am J Hum Genet. 2008 PubMed citation PMID:18304496 | PMCID:PMC2427214 pubmed 18304496 create date:2008/02/29 | first author:Kloeckener-Gruissem B

73 Localization of D-amino acid oxidase mRNA in the mouse kidney and the effect of testosterone treatment. /pubmed/8574884 Koibuchi N, Konno R, Matsuzaki S, Ohtake H, Niwa A, Yamaoka S. Histochem Cell Biol. 1995 Nov;104(5):349-55. Histochem Cell Biol. 1995 PubMed citation PMID:8574884 pubmed 8574884 create date:1995/11/01 | first author:Koibuchi N

74 The anti-apoptotic effect of regucalcin is mediated through multisignaling pathways. /pubmed/23670020 Yamaguchi M. Apoptosis. 2013 Oct;18(10):1145-53. doi: 10.1007/s10495-013-0859-x. Review. Apoptosis. 2013 PubMed citation PMID:23670020 | PMCID:PMC3775152 pubmed 23670020 create date:2013/05/15 | first author:Yamaguchi M

75 Non-type I cystinuria caused by mutations in SLC7A9, encoding a subunit (bo,+AT) of rBAT. /pubmed/10471498 Feliubadal テウ L, Font M, Purroy J, Rousaud F, Estivill X, Nunes V, Golomb E, Centola M, Aksentijevich I, Kreiss Y, Goldman B, Pras M, Kastner DL, Pras E, Gasparini P, Bisceglia L, Beccia E, Gallucci M, de Sanctis L, Ponzone A, Rizzoni GF, Zelante L, et al. Nat Genet. 1999 Sep;23(1):52-7. Nat Genet. 1999 PubMed citation PMID:10471498 pubmed 10471498 create date:1999/09/02 | first author:Feliubadal テウ L

76 SGLT2 mediates glucose reabsorption in the early proximal tubule./pubmed/20616166 Vallon V, Platt KA, Cunard R, Schroth J, Whaley J, Thomson SC, Koepsell H, Rieg T. J Am Soc Nephrol. 2011 Jan;22(1):104-12. doi: 10.1681/ASN.2010030246. Epub 2010 Jul 8. J Am Soc Nephrol. 2011 PubMed citation PMID:20616166 | PMCID:PMC3014039 pubmed 20616166 create date:2010/07/10 | first author:Vallon V

77 Cloning and expression of bovine sodium/glucose cotransporters. /pubmed/15591382 Zhao FQ, Zheng YC, Wall EH, McFadden TB. J Dairy Sci. 2005 Jan;88(1):182-94. J Dairy Sci. 2005 PubMed citation PMID:15591382 pubmed 15591382 create date:2004/12/14 | first author:Zhao FQ

78 Differential kidney proteome profiling in a murine model of renal fibrosis under treatment with mycophenolate mofetil. /pubmed/21613803 Petrova DT, Brehmer F, Schultze FC, Asif AR, Gross O, Oellerich M, Brandhorst G. Pathobiology. 2011;78(3):162-70. doi: 10.1159/000324597. Epub 2011 May 26. Pathobiology. 2011 PubMed citation PMID:21613803 pubmed 21613803 create date:2011/05/27 | first author:Petrova DT

79 Human organic anion transporters and human organic cation transporters mediate renal transport of prostaglandins. /pubmed/11907186 Kimura H, Takeda M, Narikawa S, Enomoto A, Ichida K, Endou H. J Pharmacol Exp Ther. 2002 Apr;301(1):293-8. J Pharmacol Exp Ther. 2002 PubMed citation PMID:11907186 pubmed 11907186 create date:2002/03/22 | first author:Kimura H

80 Determination of choline dehydrogenase activity along the rat nephron. /pubmed/8868068 Miller B, Schmid H, Chen TJ, Schmolke M, Guder WG. Biol Chem Hoppe Seyler. 1996 Feb;377(2):129-37. Biol Chem Hoppe Seyler. 1996 PubMed citation PMID:8868068 pubmed 8868068 create date:1996/02/01 | first author:Miller B

81 Human renal organic anion transporter 4 operates as an asymmetric urate transporter. /pubmed/17229912 Hagos Y, Stein D, Ugele B, Burckhardt G, Bahn A. J Am Soc Nephrol. 2007 Feb;18(2):430-9. Epub 2007 Jan 17. J Am Soc Nephrol. 2007 PubMed citation PMID:17229912 pubmed 17229912 create date:2007/01/19 | first author:Hagos Y

82 Plasma urate level is directly regulated by a voltage-driven urate efflux transporter URATv1 (SLC2A9) in humans. /pubmed/18701466 Anzai N, Ichida K, Jutabha P, Kimura T, Babu E, Jin CJ, Srivastava S, Kitamura K, Hisatome I, Endou H, Sakurai H. J Biol Chem. 2008 Oct 3;283(40):26834-8. doi: 10.1074/jbc.C800156200. Epub 2008 Aug 13. Erratum in: J Biol Chem. 2008 Nov 14;283(46):32152. J Biol Chem. 2008 PubMed citation PMID:18701466 pubmed 18701466 create date:2008/08/15 | first author:Anzai N

83 Formation of 20-hydroxyeicosatetraenoic acid, a vasoactive and natriuretic eicosanoid, in human kidney. Role of Cyp4F2 and Cyp4A11. /pubmed/10660572 Lasker JM, Chen WB, Wolf I, Bloswick BP, Wilson PD, Powell PK. J Biol Chem. 2000 Feb 11;275(6):4118-26. J Biol Chem. 2000 PubMed citation PMID:10660572 pubmed 10660572 create date:2000/02/08 | first author:Lasker JM

84 Immunohistochemical localization of sodium-dependent L-ascorbic acid transporter 1 protein in rat kidney. /pubmed/16673096 Lee JH, Oh CS, Mun GH, Kim JH, Chung YH, Hwang YI, Shin DH, Lee WJ. Histochem Cell Biol. 2006 Oct;126(4):491-4. Epub 2006 May 4. Histochem Cell Biol. 2006 PubMed citation PMID:16673096 pubmed 16673096 create date:2006/05/05 | first author:Lee JH

85 Evi27 encodes a novel membrane protein with homology to the IL17 receptor. /pubmed/10815801 Tian E, Sawyer JR, Largaespada DA, Jenkins NA, Copeland NG, Shaughnessy JD Jr. Oncogene. 2000 Apr 20;19(17):2098-109. Oncogene. 2000 PubMed citation PMID:10815801 pubmed 10815801 create date:2000/05/18 | first author:Tian E

86 Cloning, functional characterization, and localization of a rat renal Na+-dicarboxylate transporter. /pubmed/9691021 Sekine T, Cha SH, Hosoyamada M, Kanai Y, Watanabe N, Furuta Y, Fukuda K, Igarashi T, Endou H. Am J Physiol. 1998 Aug;275(2 Pt 2):F298-305. Am J Physiol. 1998 PubMed citation PMID:9691021 pubmed 9691021 create date:1998/08/05 | first author:Sekine T

87 Identification of UDP glycosyltransferase 3A1 as a UDP N-acetylglucosaminyltransferase. /pubmed/18981171 Mackenzie PI, Rogers A, Treloar J, Jorgensen BR, Miners JO, Meech R. J Biol Chem. 2008 Dec 26;283(52):36205-10. doi: 10.1074/jbc.M807961200. Epub 2008 Nov 3. J Biol Chem. 2008 PubMed citation PMID:18981171 | PMCID:PMC2662294 pubmed 18981171 create date:2008/11/05 | first author:Mackenzie PI

88 PAX2 activates WNT4 expression during mammalian kidney development. /pubmed/16368682 Torban E, Dziarmaga A, Iglesias D, Chu LL, Vassilieva T, Little M, Eccles M, Discenza M, Pelletier J, Goodyer P. J Biol Chem. 2006 May 5;281(18):12705-12. Epub 2005 Dec 19. J Biol Chem. 2006 PubMed citation PMID:16368682 pubmed 16368682 create date:2005/12/22 | first author:Torban E

89 Isolation, sequence identification and tissue expression profiles of 3 novel porcine genes: ASPA, NAGA, and HEXA. /pubmed/18670062 Shu X, Liu Y, Yang L, Song C, Hou J. J Appl Genet. 2008;49(3):257-61. doi: 10.1007/BF03195622. J Appl Genet. 2008 PubMed citation PMID:18670062 pubmed 18670062 create date:2008/08/02 | first author:Shu X

90 Expression of concentrative nucleoside transporters SLC28 (CNT1, CNT2, and CNT3) along the rat nephron: effect of diabetes. /pubmed/16014043 Rodr ﾃｭ guez-Mulero S, Errasti-Murugarren E, Ballar ﾃ ｭ n J, Felipe A, Doucet A, Casado FJ, Pastor-Anglada M. Kidney Int. 2005 Aug;68(2):665-72. Kidney Int. 2005 PubMed citation PMID:16014043 pubmed 16014043 create date:2005/07/15 | first author:Rodr ﾃｭ guez-Mulero S

91 Physiological oxygenation status is required for fully differentiated phenotype in kidney cortex proximal tubules. /pubmed/16597615 de Laplanche E, Gouget K, Cl ﾃｩ ris G, Dragounoff F, Demont J, Morales A, Bezin L, Godinot C, Perri ﾃｨ re G, Mouchiroud D, Simonnet H. Am J Physiol Renal Physiol. 2006 Oct;291(4):F750-60. Epub 2006 Apr 4. Am J Physiol Renal Physiol. 2006 PubMed citation PMID:16597615 pubmed 16597615 create date:2006/04/07 | first author:de Laplanche E

92 Rat brain aspartate ?-decarboxylase. A comparative study with the liver enzyme. /pubmed/20492933 Wong PT. Neurochem Int. 1985;7(2):351-5. Neurochem Int. 1985 PubMed citation PMID:20492933 pubmed 20492933 create date:1985/01/01 | first author:Wong PT

93 Peripheral distribution of kynurenine metabolites and activity of kynurenine pathway enzymes in renal failure. /pubmed/12832720 Pawlak D, Tankiewicz A, Matys T, Buczko W. J Physiol Pharmacol. 2003 Jun;54(2):175-89. J Physiol Pharmacol. 2003 PubMed citation PMID:12832720 pubmed 12832720 create date:2003/07/02 | first author:Pawlak D

94 Regulation of renin secretion by renal juxtaglomerular cells. /pubmed/22733355 Friis UG, Madsen K, Stubbe J, Hansen PB, Svenningsen P, Bie P, Sk テク tt O, Jensen BL. Pflugers Arch. 2013 Jan;465(1):25-37. doi: 10.1007/s00424-012-1126-7. Epub 2012 Jun 26. Review. Pflugers Arch. 2013 PubMed citation PMID:22733355 pubmed 22733355 create date:2012/06/27 | first author:Friis UG

95 Biochemical and molecular characterization of a novel choline-specific glycerophosphodiester phosphodiesterase belonging to the nucleotide pyrophosphatase/phosphodiesterase family. /pubmed/15788404 Sakagami H, Aoki J, Natori Y, Nishikawa K, Kakehi Y, Natori Y, Arai H. J Biol Chem. 2005 Jun 17;280(24):23084-93. Epub 2005 Mar 23. J Biol Chem. 2005 PubMed citation PMID:15788404 pubmed 15788404 create date:2005/03/25 | first author:Sakagami H

96 The betaine/GABA transporter and betaine: roles in brain, kidney, and liver. /pubmed/24795654 Kempson SA, Zhou Y, Danbolt NC. Front Physiol. 2014;5:159. doi: 10.3389/fphys.2014.00159. Review. Front Physiol. 2014 PubMed citation PMID:24795654 | PMCID:PMC4006062 pubmed 24795654 create date:2014/05/06 | first author:Kempson SA

97 Xlcaax-1 is localized to the basolateral membrane of kidney tubule and other polarized epithelia during Xenopus development. /pubmed/1537427 Cornish JA, Kloc M, Decker GL, Reddy BA, Etkin LD. Dev Biol. 1992 Mar;150(1):108-20. Dev Biol. 1992 PubMed citation PMID:1537427 pubmed 1537427 create date:1992/03/01 | first author:Cornish JA

98 Cadherin-6 is required for zebrafish nephrogenesis during early development. /pubmed/17294363 Kubota F, Murakami T, Mogi K, Yorifuji H. Int J Dev Biol. 2007;51(2):123-9. Int J Dev Biol. 2007 PubMed citation PMID:17294363 pubmed 17294363 create date:2007/02/13 | first author:Kubota F

99 The SLC34 family of sodium-dependent phosphate transporters. /pubmed/24352629 Wagner CA, Hernando N, Forster IC, Biber J. Pflugers Arch. 2014 Jan;466(1):139-53. doi: 10.1007/s00424-013-1418-6. Epub 2013 Dec 19. Review. Pflugers Arch. 2014 PubMed citation PMID:24352629 pubmed 24352629 create date:2013/12/20 | first author:Wagner CA

100 ABCC2/Abcc2: a multispecific transporter with dominant excretory functions. /pubmed/20082599 Jemnitz K, Heredi-Szabo K, Janossy J, Ioja E, Vereczkey L, Krajcsi P. Drug Metab Rev. 2010 Aug;42(3):402-36. doi: 10.3109/03602530903491741. Review. Drug Metab Rev. 2010 PubMed citation PMID:20082599 pubmed 20082599 create date:2010/01/20 | first author:Jemnitz K

101 OSTalpha-OSTbeta: a major basolateral bile acid and steroid transporter in human intestinal, renal, and biliary epithelia. /pubmed/16317684 Ballatori N, Christian WV, Lee JY, Dawson PA, Soroka CJ, Boyer JL, Madejczyk MS, Li N. Hepatology. 2005 Dec;42(6):1270-9. Hepatology. 2005 PubMed citation PMID:16317684 pubmed 16317684 create date:2005/12/01 | first author:Ballatori N

102 Hijacking solute carriers for proton-coupled drug transport. /pubmed/21186281 Anderson CM, Thwaites DT. Physiology (Bethesda). 2010 Dec;25(6):364-77. doi: 10.1152/physiol.00027.2010. Review. Physiology (Bethesda). 2010 PubMed citation PMID:21186281 pubmed 21186281 create date:2010/12/28 | first author:Anderson CM

103 Orphan transporter SLC6A18 is renal neutral amino acid transporter B0AT3. /pubmed/19478081 Singer D, Camargo SM, Huggel K, Romeo E, Danilczyk U, Kuba K, Chesnov S, Caron MG, Penninger JM, Verrey F. J Biol Chem. 2009 Jul 24;284(30):19953-60. doi: 10.1074/jbc.M109.011171. Epub 2009 May 28. J Biol Chem. 2009 PubMed citation PMID:19478081 | PMCID:PMC2740421 pubmed 19478081 create date:2009/05/30 | first author:Singer D

104 Proteomics profiling of nuclear proteins for kidney fibroblasts suggests hypoxia, meiosis, and cancer may meet in the nucleus. /pubmed/15942958 Shakib K, Norman JT, Fine LG, Brown LR, Godovac-Zimmermann J. Proteomics. 2005 Jul;5(11):2819-38. Proteomics. 2005 PubMed citation PMID:15942958 pubmed 15942958 create date:2005/06/09 | first author:Shakib K

105 Expression and functionality of the Na+/myo-inositol cotransporter SMIT2 in rabbit kidney. /pubmed/17306760 Lahjouji K, Aouameur R, Bissonnette P, Coady MJ, Bichet DG, Lapointe JY. Biochim Biophys Acta. 2007 May;1768(5):1154-9. Epub 2007 Jan 19. Biochim Biophys Acta. 2007 PubMed citation PMID:17306760 pubmed 17306760 create date:2007/02/20 | first author:Lahjouji K

106 Genetic polymorphisms in calcitonin receptor gene and risk for recurrent kidney calcium stone disease. /pubmed/24296906 Shakhssalim N, Basiri A, Houshmand M, Pakmanesh H, Golestan B, Azadvari M, Aryan H, Kashi AH. Urol Int. 2014;92(3):356-62. doi: 10.1159/000353348. Epub 2013 Nov 27. Urol Int. 2014 PubMed citation PMID:24296906 pubmed 24296906 create date:2013/12/04 | first author:Shakhssalim N

107 Insulin receptor-related receptor messenger ribonucleic acid: quantitative distribution and localization to subpopulations of epithelial cells in stomach and kidney. /pubmed/7649121 Mathi SK, Chan J, Watt VM. Endocrinology. 1995 Sep;136(9):4125-32. Endocrinology. 1995 PubMed citation PMID:7649121 pubmed 7649121 create date:1995/09/01 | first author:Mathi SK

108 Localization of Tamm-Horsfall glycoprotein in the normal rat kidney and the effect of adrenalectomy on its localization in the hamster and rat kidney. /pubmed/7130054 Sikri KL, Alexander DP, Foster CL. J Anat. 1982 Aug;135(Pt 1):29-45. J Anat. 1982 PubMed citation PMID:7130054 | PMCID:PMC1168126 pubmed 7130054 create date:1982/08/01 | first author:Sikri KL

109 Expression of the Na-K-2Cl cotransporter by macula densa and thick ascending limb cells of rat and rabbit nephron. /pubmed/8698854 Oberm テシ ller N, Kunchaparty S, Ellison DH, Bachmann S. J Clin Invest. 1996 Aug 1;98(3):635-40. J Clin Invest. 1996 PubMed citation PMID:8698854 | PMCID:PMC507472 pubmed 8698854 create date:1996/08/01 | first author:Oberm テシ ller N

110 Immunolocalization of calcitriol receptor, 24-hydroxylase cytochrome P-450, and calbindin D28k in human kidney. /pubmed/8160797 Kumar R, Schaefer J, Grande JP, Roche PC. Am J Physiol. 1994 Mar;266(3 Pt 2):F477-85. Am J Physiol. 1994 PubMed citation PMID:8160797 pubmed 8160797 create date:1994/03/01 | first author:Kumar R

111 Expression of the potassium channel ROMK in adult and fetal human kidney. /pubmed/15895241 N テシ sing RM, Pantalone F, Gr テカ ne HJ, Seyberth HW, Wegmann M. Histochem Cell Biol. 2005 Jun;123(6):553-9. Epub 2005 May 14. Histochem Cell Biol. 2005 PubMed citation PMID:15895241 pubmed 15895241 create date:2005/05/17 | first author:N テシ sing RM

112 The sodium chloride cotransporter SLC12A3: new roles in sodium, potassium, and blood pressure regulation. /pubmed/24310820 Moes AD, van der Lubbe N, Zietse R, Loffing J, Hoorn EJ. Pflugers Arch. 2014 Jan;466(1):107-18. doi: 10.1007/s00424-013-1407-9. Epub 2013 Dec 6. Review. Pflugers Arch. 2014 PubMed citation PMID:24310820 pubmed 24310820 create date:2013/12/07 | first author:Moes AD

113 Expression of claudin-7 and -8 along the mouse nephron. /pubmed/14722018 Li WY, Huey CL, Yu AS. Am J Physiol Renal Physiol. 2004 Jun;286(6):F1063-71. Epub 2004 Jan 13. Am J Physiol Renal Physiol. 2004 PubMed citation PMID:14722018 pubmed 14722018 create date:2004/01/15 | first author:Li WY

114 Calcium-sensing receptor and calcium kidney stones. /pubmed/22107799 Vezzoli G, Terranegra A, Rainone F, Arcidiacono T, Cozzolino M, Aloia A, Dogliotti E, Cusi D, Soldati L. J Transl Med. 2011 Nov 22;9:201. doi: 10.1186/1479-5876-9-201. Review. J Transl Med. 2011 PubMed citation PMID:22107799 | PMCID:PMC3339356 pubmed 22107799 create date:2011/11/24 | first author:Vezzoli G

115 Cloning and expression of glycolipid transfer protein from bovine and porcine brain. /pubmed/10671554 Lin X, Mattjus P, Pike HM, Windebank AJ, Brown RE. J Biol Chem. 2000 Feb 18;275(7):5104-10. J Biol Chem. 2000 PubMed citation PMID:10671554 | PMCID:PMC2621014 pubmed 10671554 create date:2000/02/15 | first author:Lin X

116 Comparative analysis of AP-2 alpha and AP-2 beta gene expression during murine embryogenesis. /pubmed/8989526 Moser M, R ﾃｼ schoff J, Buettner R. Dev Dyn. 1997 Jan;208(1):115-24. Dev Dyn. 1997 PubMed citation PMID:8989526 pubmed 8989526 create date:1997/01/01 | first author:Moser M

117 New insights into regulated aquaporin-2 function. /pubmed/23852332 Fenton RA, Pedersen CN, Moeller HB. Curr Opin Nephrol Hypertens. 2013 Sep;22(5):551-8. doi: 10.1097/MNH.0b013e328364000d. Review. Curr Opin Nephrol Hypertens. 2013 PubMed citation PMID:23852332 pubmed 23852332 create date:2013/07/16 | first author:Fenton RA

118 Hepsin, a cell membrane-associated protease. Characterization, tissue distribution, and gene localization. /pubmed/1885621 Tsuji A, Torres-Rosado A, Arai T, Le Beau MM, Lemons RS, Chou SH, Kurachi K. J Biol Chem. 1991 Sep 5;266(25):16948-53. J Biol Chem. 1991 PubMed citation PMID:1885621 pubmed 1885621 create date:1991/09/05 | first author:Tsuji A

119 Molecular cloning and characterization of novel tissue-specific isoforms of the human vacuolar H(+)-ATPase C, G and d subunits, and their evaluation in autosomal recessive distal renal tubular acidosis. /pubmed/12384298 Smith AN, Borthwick KJ, Karet FE. Gene. 2002 Sep 4;297(1-2):169-77. Gene. 2002 PubMed citation PMID:12384298 pubmed 12384298 create date:2002/10/18 | first author:Smith AN

120 Parvalbumin: calcium and magnesium buffering in the distal nephron. /pubmed/23144069 Olinger E, Schwaller B, Loffing J, Gailly P, Devuyst O. Nephrol Dial Transplant. 2012 Nov;27(11):3988-94. doi: 10.1093/ndt/gfs457. Review. Nephrol Dial Transplant. 2012 PubMed citation PMID:23144069 pubmed 23144069 create date:2012/11/13 | first author:Olinger E

121 Effect of collecting duct-specific deletion of both Rh B Glycoprotein (Rhbg) and Rh C Glycoprotein (Rhcg) on renal response to metabolic acidosis. /pubmed/24338819 Lee HW, Verlander JW, Handlogten ME, Han KH, Weiner ID. Am J Physiol Renal Physiol. 2014 Feb 15;306(4):F389-400. doi: 10.1152/ajprenal.00176.2013. Epub 2013 Dec 11. Am J Physiol Renal Physiol. 2014 PubMed citation PMID:24338819 | PMCID:PMC3920025 pubmed 24338819 create date:2013/12/18 | first author:Lee HW

122 AE4 is a DIDS-sensitive Cl(-)/HCO(-)(3) exchanger in the basolateral membrane of the renal CCD and the SMG duct. /pubmed/12225984 Ko SB, Luo X, Hager H, Rojek A, Choi JY, Licht C, Suzuki M, Muallem S, Nielsen S, Ishibashi K. Am J Physiol Cell Physiol. 2002 Oct;283(4):C1206-18. Am J Physiol Cell Physiol. 2002 PubMed citation PMID:12225984 pubmed 12225984 create date:2002/09/13 | first author:Ko SB
