# Supplementary material for: The Kidney Transcriptome and Proteome Defined by Transcriptomics and Antibody-Based Profiling
Source: PLoS One. 2014 Dec 31;9(12):e116125. doi: 10.1371/journal.pone.0116125 (PMC4281243; doi:10.1371/journal.pone.0116125)
Supplement: S5 Table — GO analyses of the different nephron segment and collecting duct specific proteins. (PDF) [file pone.0116125.s007.pdf]

Table S5. GO analysis of the different nephron segment and collecting duct specific proteins/proteomes

Segment	Description top10 (biological process)	GO Term	Enrichment	Description top10 (molecular function)	GO Term	Enrichment	Description top10 (cellular component)	GO Term	Enrichment
Glomeruli	regulation of microvillus assembly	GO:0032534	1457.33	myo-inositol transmembrane transporter activity	GO:0005365	1457.33	filtration diaphragm	GO:0036056	624.57
	slit diaphragm assembly	GO:0036060	1457.33	myo-inositol:sodium symporter activity	GO:0005367	1457.33	slit diaphragm	GO:0036057	624.57
	filtration diaphragm assembly	GO:0036058	1457.33				membrane raft	GO:0045121	22.54
	branch elongation involved in ureteric bud branching	GO:0060681	1457.33				cell-cell junction	GO:0005911	15.39
	glomerular visceral epithelial cell development	GO:0072015	546.5				integral component of plasma membrane	GO:0005887	8.38
	glomerular epithelial cell development	GO:0072310	485.78				plasma membrane	GO:0005886	3.38
	excretion	GO:0007588	66.24				integral component of membrane	GO:0016021	3.02
	epithelial cell development	GO:0002064	60.72				membrane part	GO:0044425	2.4
Proximal tubule	urate transport	GO:0015747	149.47	phosphoenolpyruvate carboxykinase activity	GO:0004611	149.47	brush border membrane	GO:0031526	21.87
	fructose catabolic process	GO:0006001	149.47	phosphoenolpyruvate carboxykinase (GTP) activity	GO:0004613	149.47	apical plasma membrane	GO:0016324	9.83
	amino-acid betaine catabolic process	GO:0006579	99.65	betaine-homocysteine S-methyltransferase activity	GO:0047150	149.47	basolateral plasma membrane	GO:0016323	6.06
	NADH oxidation	GO:0006116	99.65	urate transmembrane transporter activity	GO:0015143	149.47	cell projection membrane	GO:0031253	5.34
	quinolinate metabolic process	GO:0046874	74.74	salt transmembrane transporter activity	GO:1901702	149.47	mitochondrial matrix	GO:0005759	4.55
	L-cystine transport	GO:0015811	74.74	homocysteine S-methyltransferase activity	GO:0008898	99.65	integral component of plasma membrane	GO:0005887	3.73
	pyridine-containing compound catabolic process	GO:0072526	74.74	aminoacylase activity	GO:0004046	89.68	intracellular organelle lumen	GO:0070013	3.22
	response to methotrexate	GO:0031427	74.74	glycine N-acyltransferase activity	GO:0047961	74.74	organelle lumen	GO:0043233	3.15
	sulfur amino acid transport	GO:0000101	74.74	L-cystine transmembrane transporter activity	GO:0015184	74.74	membrane-enclosed lumen	GO:0031974	3.12
	amino-acid betaine biosynthetic process	GO:0006578	64.06	sodium-independent organic anion transmembrane transporter activity	GO:0015347	59.79	mitochondrial part	GO:0044429	2.89
Distal tubule	regulation of G-protein activated inward rectifier potassium channel activity	GO:1900128	1943.11	sodium:chloride symporter activity	GO:0015378	1943.11	No GO enrichment found		
	metanephric connecting tubule development	GO:0072286	1943.11	cation:chloride symporter activity	GO:0015377	485.78			
	connecting tubule development	GO:0072027	1943.11	anion:cation symporter activity	GO:0015296	80.96			
	cellular urea homeostasis	GO:0097277	1943.11	chloride transmembrane transporter activity	GO:0015108	51.82			
	cellular creatinine homeostasis	GO:0097276	1943.11	solute:cation symporter activity	GO:0015294	47.98			
	cellular ammonia homeostasis	GO:0097275	1943.11	**substrate-specific transporter activity**	**GO:0022892**	8.3			
	ammonia homeostasis	GO:0097272	1943.11						
	creatinine homeostasis	GO:0097273	1943.11						
	urea homeostasis	GO:0097274	1943.11						
	metanephric distal convoluted tubule development	GO:0072221	971.56						
Collecting duct	basement membrane disassembly	GO:0034769	2186	monovalent inorganic cation transmembrane transporter activity	GO:0015077	20.18	cytoplasmic vesicle membrane	GO:0030659	18.17
	monovalent inorganic cation transport	GO:0015672	22.08	inorganic cation transmembrane transporter activity	GO:0022890	13.92	vesicle membrane	GO:0012506	17.63
	transmembrane transport	GO:0055085	13	substrate-specific transmembrane transporter activity	GO:0022891	13.48			
	ion transport	GO:0006811	8.82	transmembrane transporter activity	GO:0022857	12.36			
				**substrate-specific transporter activity**	**GO:0022892**	11.66			
				ion transmembrane transporter activity	GO:0015075	11.61			
				transporter activity	GO:0005215	9.5			

Only 1 overlap GO term among each nephron segments.
