# Supplementary material for: The Kidney Transcriptome and Proteome Defined by Transcriptomics and Antibody-Based Profiling
Source: PLoS One. 2014 Dec 31;9(12):e116125. doi: 10.1371/journal.pone.0116125 (PMC4281243; doi:10.1371/journal.pone.0116125)
Supplement: S6 Table — GO analyses of the group enriched proteins. (PDF) [file pone.0116125.s008.pdf]

Table S6. GO analysis of 39 liver/kidney shared proteins

Gene category	Description top10 (biological process)	GO Term	Enrichment	Description top10 (molecular function)	GO Term	Enrichment	Description top10 (cellular component)	GO Term	Enrichment
Group enriched genes (shared only liver) n=39	amino-acid betaine catabolic process	GO:0006579	298.92	**betaine-homocysteine S-methyltransferase activity**	**GO:0047150**	**448.38**	microbody	GO:0042579	19.49
	beta-alanine metabolic process	GO:0019482	298.92	homocysteine S-methyltransferase activity	GO:0008898	298.92	peroxisome	GO:0005777	19.29
	proline catabolic process	GO:0006562	224.19	arachidonic acid omega-hydroxylase activity	GO:0052869	298.92	organelle lumen	GO:0043233	5.82
	alanine catabolic process	GO:0006524	179.35	**alkane 1-monooxygenase activity**	**GO:0018685**	**224.19**	intracellular organelle lumen	GO:0070013	5.63
	alanine metabolic process	GO:0006522	179.35	oxidoreductase activity, acting on paired donors, with incorporation or reduction of molecular oxygen, reduced iron-sulfur protein as one donor, and incorporation of one atom of oxygen	GO:0016713	128.11	membrane-enclosed lumen	GO:0031974	5.35
	pyruvate family amino acid catabolic process	GO:0009080	179.35	S-methyltransferase activity	GO:0008172	128.11	cytoplasmic part	GO:0044444	1.96
	pyruvate family amino acid metabolic process	GO:0009078	179.35	butyrate-CoA ligase activity	GO:0047760	112.1			
	glyoxylate metabolic process	GO:0046487	112.1	acid-thiol ligase activity	GO:0016878	67.26			
	proline metabolic process	GO:0006560	89.68	phosphate ion transmembrane transporter activity	GO:0015114	64.05			
	cellular glucuronidation	GO:0052695	84.07	oxidoreductase activity, acting on the CH-NH group of donors	GO:0016645	53.81			
Group enriched genes (except shared only liver) n=117	**fructose catabolic process**	**GO:0006001**	**158.97**	inward rectifier potassium channel activity	GO:0005242	22.71	**brush border membrane**	**GO:0031526**	**15.51**
	NADH oxidation	GO:0006116	105.98	organic acid:sodium symporter activity	GO:0005343	17.03	basolateral plasma membrane	GO:0016323	9.67
	potassium ion homeostasis	GO:0055075	52.99	solute:sodium symporter activity	GO:0015370	16.22	**apical plasma membrane**	**GO:0016324**	**9.06**
	renal water homeostasis	GO:0003091	52.99	anion:cation symporter activity	GO:0015296	13.25	cell projection part	GO:0044463	3.08
	G1 to G0 transition	GO:0070314	45.42	solute:cation symporter activity	GO:0015294	11.78	plasma membrane part	GO:0044459	2.84
	fat-soluble vitamin biosynthetic process	GO:0042362	45.42	sodium ion transmembrane transporter activity	GO:0015081	11.54	cell projection	GO:0042995	2.79
	spinal cord motor neuron cell fate specification	GO:0021520	45.42	antiporter activity	GO:0015297	10.78	integral component of plasma membrane	GO:0005887	2.74
	cellular anion homeostasis	GO:0030002	43.36	secondary active transmembrane transporter activity	GO:0015291	10.26	plasma membrane	GO:0005886	2.07
	fructose metabolic process	GO:0006000	43.36	symporter activity	GO:0015293	10.01	integral component of membrane	GO:0016021	1.58
	vitamin D metabolic process	GO:0042359	43.36	organic anion transmembrane transporter activity	GO:0008514	8.76	membrane part	GO:0044425	1.54
kidney enriched n=387	urate transport	GO:0015747	48.85	arachidonic acid omega-hydroxylase activity	GO:0052869	48.85	vacuolar proton-transporting V-type ATPase complex	GO:0016471	21.71
	**fructose catabolic process**	**GO:0006001**	**48.85**	phosphoenolpyruvate carboxykinase activity	GO:0004611	48.85	proton-transporting V-type ATPase complex	GO:0033176	21.71
	mesonephric duct formation	GO:0072181	48.85	phosphoenolpyruvate carboxykinase (GTP) activity	GO:0004613	48.85	filtration diaphragm	GO:0036056	20.94
	metanephric thick ascending limb development	GO:0072233	48.85	parathyroid hormone receptor activity	GO:0004991	48.85	slit diaphragm	GO:0036057	20.94
	thick ascending limb development	GO:0072023	48.85	**betaine-homocysteine S-methyltransferase activity**	**GO:0047150**	**48.85**	**brush border membrane**	**GO:0031526**	**11.91**
	nephric duct formation	GO:0072179	36.64	urate transmembrane transporter activity	GO:0015143	48.85	brush border	GO:0005903	10.18
	mesonephric tubule formation	GO:0072172	36.64	salt transmembrane transporter activity	GO:1901702	48.85	**apical plasma membrane**	**GO:0016324**	**7.71**
	metanephric distal convoluted tubule development	GO:0072221	36.64	oxidoreductase activity, acting on the CH-NH group of donors, flavin as acceptor	GO:0046997	48.85	basolateral plasma membrane	GO:0016323	7.26
	distal convoluted tubule development	GO:0072025	36.64	**alkane 1-monooxygenase activity**	**GO:0018685**	**36.64**	apical part of cell	GO:0045177	5.78
	response to methotrexate	GO:0031427	36.64	aminoacylase activity	GO:0004046	29.31	cell projection membrane	GO:0031253	3.49

Only 1 overlap GO term among each nephron segments.
